# Control over the morphology and segregation of Zebrafish germ cell granules during embryonic development

**DOI:** 10.1186/1471-213X-8-58

**Published:** 2008-05-28

**Authors:** Markus J Strasser, Natalia C Mackenzie, Karin Dumstrei, La-Iad Nakkrasae, Jürg Stebler, Erez Raz

**Affiliations:** 1 Germ Cell Development, Max Planck Institute for Biophysical Chemistry, Am Fassberg 11, 37077 Göttingen, Germany; 2 Department of Biology, Faculty of Science, Mahidol University, Bangkok, Thailand; 3 Institute of Cell Biology, ZMBE, University of Münster, Von-Esmarch-Straße 56, 48149 Münster, Germany

## Abstract

**Background:**

Zebrafish germ cells contain granular-like structures, organized around the cell nucleus. These structures share common features with polar granules in Drosophila, germinal granules in Xenopus and chromatoid bodies in mice germ cells, such as the localization of the zebrafish Vasa, Piwi and Nanos proteins, among others. Little is known about the structure of these granules as well as their segregation in mitosis during early germ-cell development.

**Results:**

Using transgenic fish expressing a fluorescently labeled novel component of Zebrafish germ cell granules termed Granulito, we followed the morphology and distribution of the granules. We show that whereas these granules initially exhibit a wide size variation, by the end of the first day of development they become a homogeneous population of medium size granules. We investigated this resizing event and demonstrated the role of microtubules and the minus-end microtubule dependent motor protein Dynein in the process. Last, we show that the function of the germ cell granule resident protein the Tudor domain containing protein-7 (Tdrd7) is required for determination of granule morphology and number.

**Conclusion:**

Our results suggest that Zebrafish germ cell granules undergo a transformation process, which involves germ cell specific proteins as well as the microtubular network.

## Background

Primordial germ cells (PGCs) are progenitor cells that migrate from their site of specification to the site of the developing gonad where they differentiate into the gametes, sperm and egg. PGCs are normally specified during early development either by inductive cues (in mammals and in Urodele amphibians) [1] or by inheritance of cytoplasmic determinants (e.g. in *Drosophila, C. elegans*, zebrafish and *Xenopus*). These cytoplasmic determinants are comprised of electron dense material containing maternal RNAs and proteins, collectively termed germ plasm [2,3]. Transplantation experiments in *Drosophila *demonstrated that the germ plasm is sufficient for the induction of the germ cell fate [4]. Similarly, in zebrafish it has been shown that removal of germ plasm leads to loss of germ cell specification [5]. In zebrafish, the germ plasm is localized to the first and second cleavage furrows of the early embryo and this localization depends on the function of actin and microtubules [6,7]. At the 32-cell stage the germ plasm is incorporated into four cells that become the progenitors of the germ cell lineage [8]. During division of these progenitor cells, the germ plasm is inherited by only one daughter cell [9,10], thereby maintaining the number of presumptive germ cells constant. However, after 4 hours of zebrafish development the germ plasm is segregated symmetrically, a change that coincides with an increase in PGC numbers. Later in development, Zebrafish germ cells contain unique granular structures organized around the germ cell nucleus. Several germ cell specific proteins, whose RNA is initially localized to the germ plasm, localize to these granules (e.g. Vasa [9], Nanos [11] and Dead end [12]). Germ cell specific granules are a common feature of germ cells in metazoans, including species where germ cells are specified by induction [1,13]. Although the precise function of these granular structures in germ cell development remains largely unknown, several RNAs and proteins localized to these structures were found to be essential for germ cell specification, survival and later development. One common marker for germ cell granules is the RNA helicase Vasa protein, which shows high sequence homology to the translation initiation factor eIF-4A [14-16]. Interestingly, while in *Drosophila *Vasa protein localizes early on to the germ plasm, in Zebrafish only the *vasa *RNA localizes to the germ plasm, while the protein is dispersed in the early embryo. Presumably only zygotically produced Vasa is later restricted to the germ line, where it localizes in granules around the germ cell nucleus [9]. In contrast to *vasa *for which no function in zebrafish PGC development has yet been attributed, other components of the Zebrafish germ cell granules, such as the RNA-binding proteins Dead end (Dnd) and Nanos1 (Nos1) are essential for germ-cell survival and maintenance of oocyte production [11,12,17].

Zebrafish germ cell granules are organized around the nucleus after the cells arrive at the region of the gonad [9,18]. Little is known however about their distribution and segregation during the first 24 hpf of Zebrafish embryonic development, in particular during cell division. Understanding these processes would contribute to the understanding of the mechanisms that promote germ cell fate maintenance and those allowing for an increase in germ cell number (e.g. during the time of germ-cell migration).

Accurate inheritance of organelles and cytoplasmic components is a key requirement for symmetrical cell division. Current models of organelle distribution suggest two distinct partitioning strategies: a stochastic partition or an ordered one [19]. Stochastic mechanisms rely on random distribution of multiple organelle copies that would provide each daughter cell with sufficient copies of the organelle, but not necessarily with the same total number [19]. For example, the large cytoplasmic area covered by the ER in mammalian cells ensures that both daughter cells inherit sufficient amount of ER that is adequately facilitated by a stochastic mechanism [20]. The chromosome distribution during cell division on the other hand, proceeds in an ordered manner in a process that involves the mitotic spindle machinery. Central players in the ordered organization and segregation of cellular components during the cell cycle are the microtubule-dependent molecular motors such as Dynein and Kinesin [21].

In this study we show that at the onset of germ cell development, zebrafish germ cell granules exhibit a strong variation of size. This appearance changes however during the first day of embryonic development when germ-cell granules become homogenous in size. The change in granule size and granule segregation to daughter cells during mitosis appears ordered and depends on the microtubule network, the function of the molecular motor protein Dynein, as well as the tudor domain-containing protein, Tdrd7.

## Results

### Identification of the novel germ cell marker granulito

**Figure 1:**
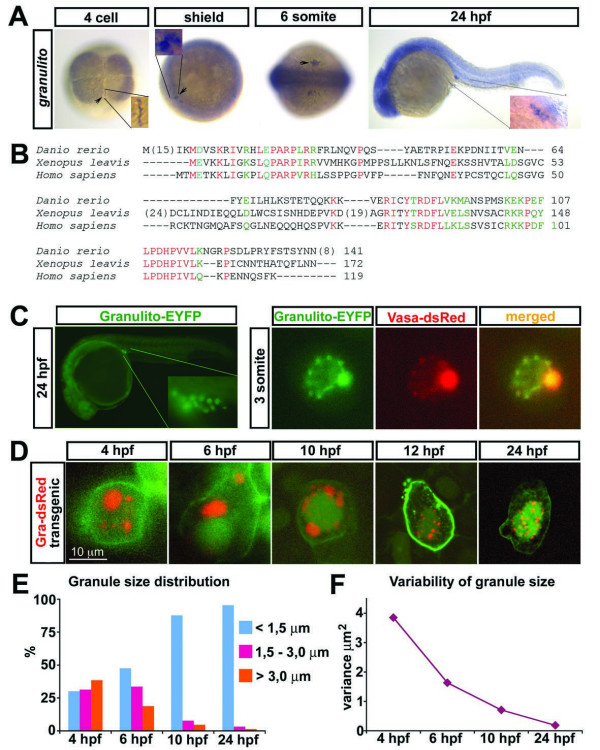
**Expression pattern of *granulito *RNA and subcellular localization of the Ganulito protein**. A) Whole-mount *in situ *hybridizations using *granulito *antisense RNA probe at the indicated stages. *granulito *is enriched at the region where the germ plasm resides (cleavage furrows, arrowheads) and is expressed in the primordial germ cells at later stages (arrowheads). B) Alignment of the zebrafish Granulito protein with those from *Xenopus leavis *and *Homo sapiens*. Red signifies conservation in all 3 species, green labels conserved substitutions. C) Subcellular localization of Granulito-EYFP. Granulito localizes specifically to germ cell granules as it colocalizes with Vasa protein. D) Germ cells of *kop-granulito-dsRedex-nos1*-3'UTR transgenic fish whose membrane and nucleus are labeled in green (except for the 4 hours stage where the nucleus is not labeled). Initially, germ cell granules with a large variety of sizes are observed. As development proceeds, the variation in germ cell granule size decreases. E) Granule size distribution at different stages. F) Variance of granule size at different stages. N >100 granules for each time point.

A novel germ cell marker, which we named *granulito *(*gra*) was identified in a microarray-based screen where the transcripts of isolated wildtype PGCs were compared to the ones in which the function of *dead end *(*dnd*), a gene essential for PGC survival [12] was knocked down. *granulito *was found to be expressed at very high levels in wild-type germ cells as compared to germ cells in which dead end was knocked down. *granulito *mRNA is enriched in the germ plasm at the 4-cell stage and is expressed in PGCs until the end of the first day of development (Figure 1A and data not shown). This expression pattern in the germ cells is similar to that of previously described germ cell markers such as *dead end *and *nanos1 *[11,12], but differs from those in that it is expressed at significant levels in somatic cells as well. *granulito *mRNA encodes a 141 amino acid protein that exhibits significant homology only to proteins in the vertebrata (Figure 1B), does not contain known protein domains and does not exhibit significant similarity to other genes. Granulito-EYFP fusion protein is localized to germ cell granules in PGCs, the structure in which the Vasa protein is found (Figure 1C). To increase the confidence in the observed localization of the fusion protein, the fluorescent protein was fused both 3' and 5' of the Granulito protein, HA and FLAG tages were generated and the open reading frame of the fusion was linked to its own 3'UTR leading to the same result (data not shown). To determine whether Granulito plays a role in zebrafish PGC development, we knocked down its function using antisense morpholino oligonucleotides. While injection of antisense oligonucleotides directed against the 5' sequence of *granulito *efficiently blocked translation of *granulito-eyfp-nos1*-3'UTR (see Additional file 1), germ cell development proceeded normally at least until 24 hours post fertilization (hpf). When high amount of antisense oligonucleotides was injected, embryonic development was arrested during gastrulation, while germ cell development appeared unaffected (data not shown). We therefore conclude that if this protein plays a role in early PGC development, its function is either redundant with that of another gene product, or that maternally provided Granulito protein (which is not affected by the antisense oligonucleotides treatment) is sufficient for performing the early role of the protein. Despite the apparent lack of function for the protein, the localization of Granulito to the Zebrafish germ cell granules could serve as a tool for investigating these structures during early stages of PGC development.

### Dynamic variation of germ cell granule size during early embryonic development

Germ cell granules in live zebrafish embryos were previously visualized by injection of the GFP fusion constructs *nanos1-gfp-nos3'UTR*, *deadend-gfp-nos3'UTR *and *vasa-gfp-nos3'UTR *[11,12,22]. However, mRNA injection does not allow adequate visualization of granules before 6 hpf due to high fluorescent background in somatic cells and poor specific signal in primordial germ cells. Additionally, Nanos1-GFP and Dead end-GFP localization appears to be diffused at this stage and not unique to granules. In order to visualize the granules at an earlier developmental stage, we generated transgenic fish expressing *granulito-dsRedEx-nos1-*3'UTR under the control of the *askopos *promoter [23]. In progeny of transgenic females expressing *egfp-nos1-*3'UTR fusion under the control of the maternal promoter of *askopos*, germ cells could be visualized as early as 3 hpf. The localization and specific translation of *granulito-dsRedEx-nos1-*3'UTR in PGCs allowed us to follow germinal granules from 4 hpf in live embryos (Figure 1D). At this stage and at 6 hpf, in most germ cells a single to few large granules were observed along with several smaller ones (Figure 1D,E). As development proceeds, the proportion of small granules increases (Figure 1D,E). Finally at 24 hpf, as the germ cells are located where the gonad will develop, most PGCs show a uniform distribution of granules around the nucleus with similar small sizes. This strong reduction in granule size variation within the first 24 hours of development is presented in Figure 1F.

Given the critical role germ cell granules components play in PGC development, the question of the control over the morphology and distribution of these structures is a central one for understanding the development of PGCs. We therefore followed the granules during cell division in different developmental stages and examined the possible role cytoskeleton components and granule-specific proteins could play in this process.

### Dynamic localization of Zebrafish germ cell granules

To elucidate the mechanism by which germ cell granules are distributed between daughter cells during mitosis, we analyzed this process during germ cell division in 10 hpf zebrafish embryos. To that end, we labeled germ cell granules using Vasa-DsRed, the nuclear envelope using LaminB2-GFP and the nuclear pores using NUPL1-GFP. We monitored nuclear envelope break down (NEBD) and granule segregation during cell division using confocal time-lapse microscopy.

**Figure 2:**
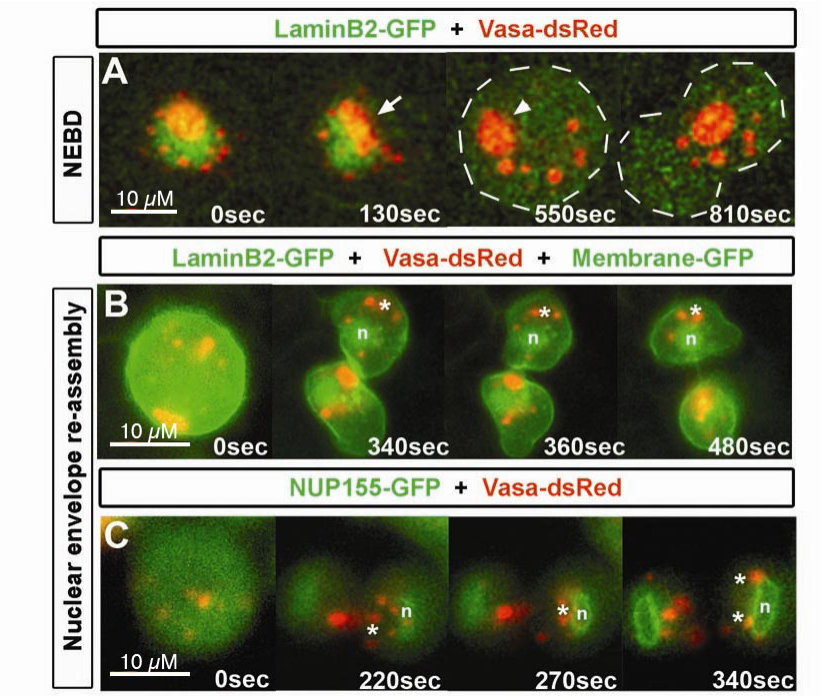
**Germ cell granule behavior during cell division in live embryos**. A) During interphase and before nuclear envelope breakdown (NEBD), the granules (labeled with Vasa-dsRed) show an organized localization around the nucleus (visualized with LaminB2-GFP). During early prophase, the nuclear envelop disassembles and germ cell granules change their shape (arrow). Once the envelope is completely disassembled granules that changed their shape readopt their spherical shape (arrowhead). All granules spread in the cytoplasm and are segregated into daughter cells. B) After cytokinesis, the nuclear envelope reassembles (n) and a group of granules spread in the cytoplasm readopt perinuclear localization (asterisk). Granules are labeled with Vasa-dsRed, nuclear envelope with LaminB2-GFP, and plasma membrane with farnesylated GFP. C) Movement of granules towards the nuclear envelope labeled with NUP155-GFP and visualized by confocal microscopy. Granules move directly (asterisk) to the area where the nuclear envelope is reassembling (n) during cytokinesis. All movies were recorded in 10 hpf embryos.

At 10 hpf, germ cell granules of diverse sizes are organized around the nuclear envelope of germ cells at interphase (Figure 2A, arrow). At the onset of cell division, during early prophase, the nuclear envelope disassembles and the granules lose their perinuclear localization and scatter throughout the cytoplasm (Figure 2A, arrowhead). Granules may appear to transiently change their shape during nuclear envelope reorganization and recover their spherical shape when detached completely from the envelope during metaphase (Figure 2A arrow).

At the end of cytokinesis, granules move directly towards the forming nuclear envelope (Figure 2B, n shows the nucleus and asterisks depict granules regaining perinuclear localization, see movie in Additional file 2). Granular movement takes place after the nuclear envelope has reassembled as visualized by the reformation of the Lamin B2 network and the accumulation of the nuclear pore complex component NUP155 (asterisks in Figure 2B and 2C).

These results suggest that the spherical shape of granules is not dependent on the nuclear envelope integrity. Moreover, during nuclear envelope re-assembly, all of the granules regain their perinuclear localization.

### Ordered segregation of germ cell granules to daughter cells during mitosis

To investigate the partition of germ cell granules during cell division, we analyzed their distribution within the two daughter cells after mitosis. In all 13 cells undergoing mitosis that were analyzed, the granule distribution among daughter cells was equal for even numbers of granules or ± 1 granule for odd number of granules (see Additional file 3 for table). However, the amount of granule material was not necessarily equally distributed. We therefore concluded that segregation of the granules in zebrafish germ cells during mitosis is not a random process but rather a regulated event that ensures segregation of similar units of this important structure.

### Localization of germ cell granules with respect to microtubules

The process of germ plasm assembly during oogenesis has been studied extensively in invertebrates. In *Drosophila*, the assembly of germ plasm is dependent on the localization of *oskar m*RNA to the posterior pole of the oocyte, a process that requires microtubule function [24]. Similarly, zebrafish germ plasm assembly occurs during the first hours following fertilization and involves microtubules as well [7]. To determine whether proper localization of germ cell granules at later stages of development depends on microtubules as well, we examined the positioning of microtubules relatisve to the germ cell granules during interphase and mitosis.

**Figure 3:**
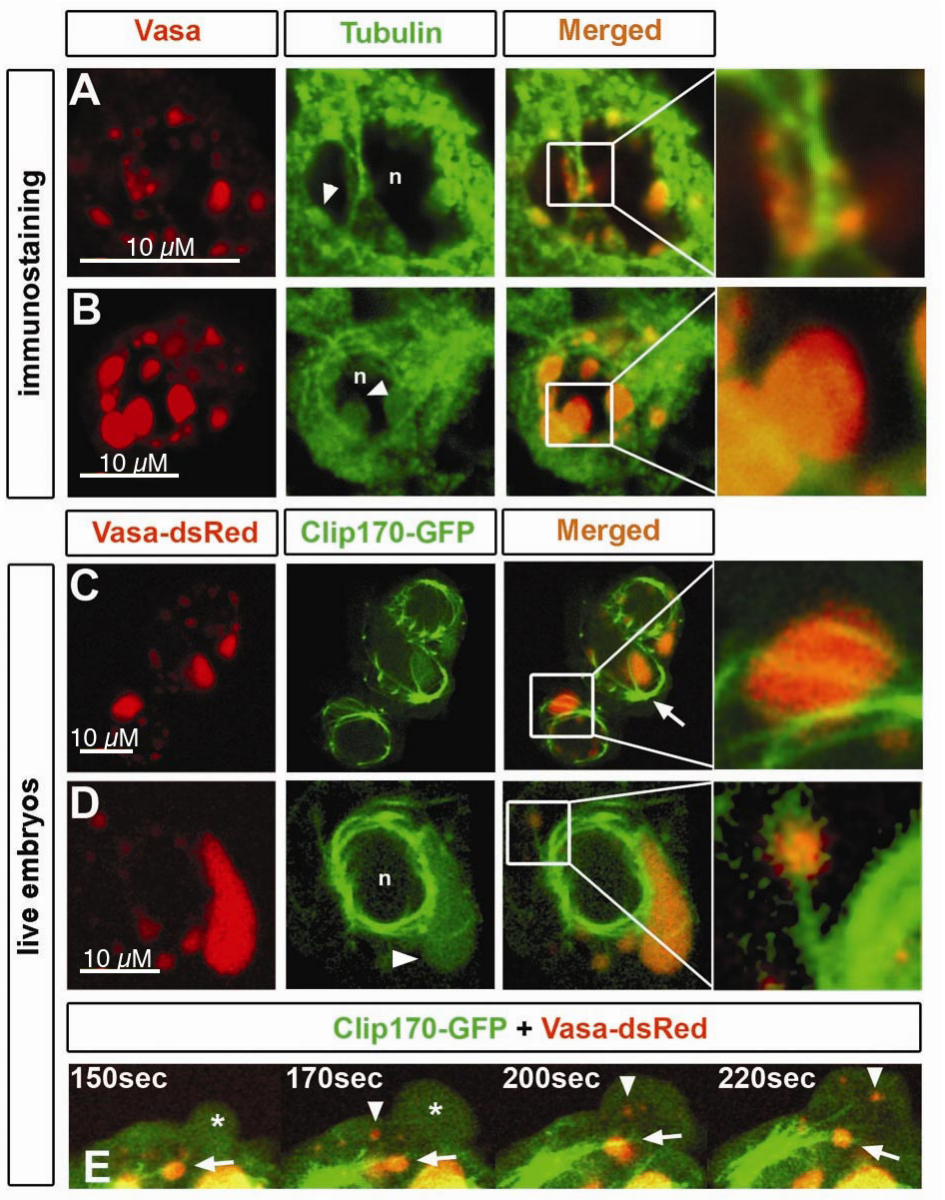
**Microtubules colocalize with germ cell granules**. A) Immunostaining of Vasa protein and α-Tubulin in germ cells of 6 hpf embryos. Perinuclear granules attached to microtubules are shown in detail. n and arrowhead depicts the nucleus and granules expressing Tubulin, respectively. B) At 16 hpf, germ cell perinuclear granules show expression of α-Tubulin within their structure (arrowhead). Nucleus is depicted by n. Pinhole value is 1.22. C).*Germ cells in *live 11 hpf embryos have a microtubular cage surrounding the nuclei, where microtubules originated from the MTOC (arrow) span the granules (detail). Microtubules were labeled with Clip170- GFP, and granules with Vasa-dsRed. D) Cytoplasmic granules colocalize with microtubular fibers projecting from the nuclear cage (detail). Tubulin expression is also found within the granule (arrowhead) of 11 hpf embryos. Nucleus is depicted by n. E) A close-up view of a germ cell of a 11 hpf embryos reveals flow of cytoplasmic granules not associated with microtubules (arrowhead) into the protrusion (asterisk) while others remain stable in colocalization with microtubules projected from the MTOC (arrow).

During interphase, germ cell granules appeared to be adjacent to microtubular structures (Figure 3A). Furthermore, α-Tubulin staining could be observed closely localized near germ cell granules (Figure 3A,B,D arrowheads in tubulin panel, n depicts nucleus).

To investigate the relationship between microtubules and granule localization in live embryos, we performed time-lapse confocal microscopy of germ cells expressing Clip170-GFP fusion protein [25]. In this analysis we found that a cage of microtubules, which originates from the microtubule-organizing center (MTOC) (Figure 3C arrow) is formed around the germ cell nucleus. Microtubules appear to traverse perinuclear granules (Figure 3C, detail) and can project towards more distant granules (Figure 3D, detail). Interestingly, we observed that some distant germ cell granules do not appear to be associated with microtubules (Figure 3E, arrowheads) and as such, flow with the cytoplasmic stream into newly forming protrusions (Figure 3E, asterisk). In contrast, microtubule-associated granules remain fixed in the vicinity of the nucleus (Figure 3E arrows). These observations are consistent with the idea that during interphase the association of the perinuclear granules with microtubular structures serves to anchor the granules in proximity to the nucleus, where they presumably exert their function.

**Figure 4:**
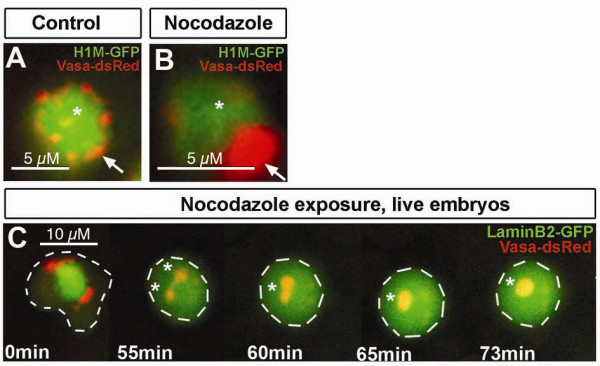
**Granules structure is affected after microtubule disruption**. A) Confocal section of germ cells in control embryos exposed to DMSO for 6 hours. Germ cell granules show normal perinuclear localization and distribution into small structures (arrow). B) Confocal section of germ cells in embryos exposed to 1 μg/ml of nocodazole for 6 hours. Nocodazole treated cells exhibit a conglomeration of Vasa positive granules forming a large granule (arrow). C) Embryos exposed to same conditions mentioned above. Vasa-dsRed labels granules, LaminB2-GFP labels nuclear envelop. The experiment shows how after laminB2 network disassembles, individual granules fuse forming bigger structures (asterisk). Cell outline is depicted with white dashed lines. All experiments were done in 11 hpf embryos.

Consistent with this notion is the finding that granule structure and localization are affected when microtubule depolarization is induced. Specifically, exposure of zebrafish embryos expressing H1M-GFP (which labels the chromatin [26]) along with Vasa-dsRed to nocodazole leads to aggregation of Vasa-positive granules resulting in enlarged structures (Figure 4B arrow). Control cells on the other hand, that are exposed to the solvent alone exhibit normal perinuclear distribution characteristic of that stage (Figure 4A arrow). Time-lapse microscopy of embryos expressing LaminB2-GFP and Vasa-dsRed treated with nocodazole revealed that following exposure to the drug granules fuse forming larger structures (Figure 4C, asterisks). Such phenotype resulted from the complete disruption of microtubular structures, as judged by staining of microtubules and by loss of cell polarity needed for migration and motility (data not shown). These results suggest that microtubules and possibly nuclear envelope components are needed for proper structural maintenance of individual granules.

**Figure 5:**
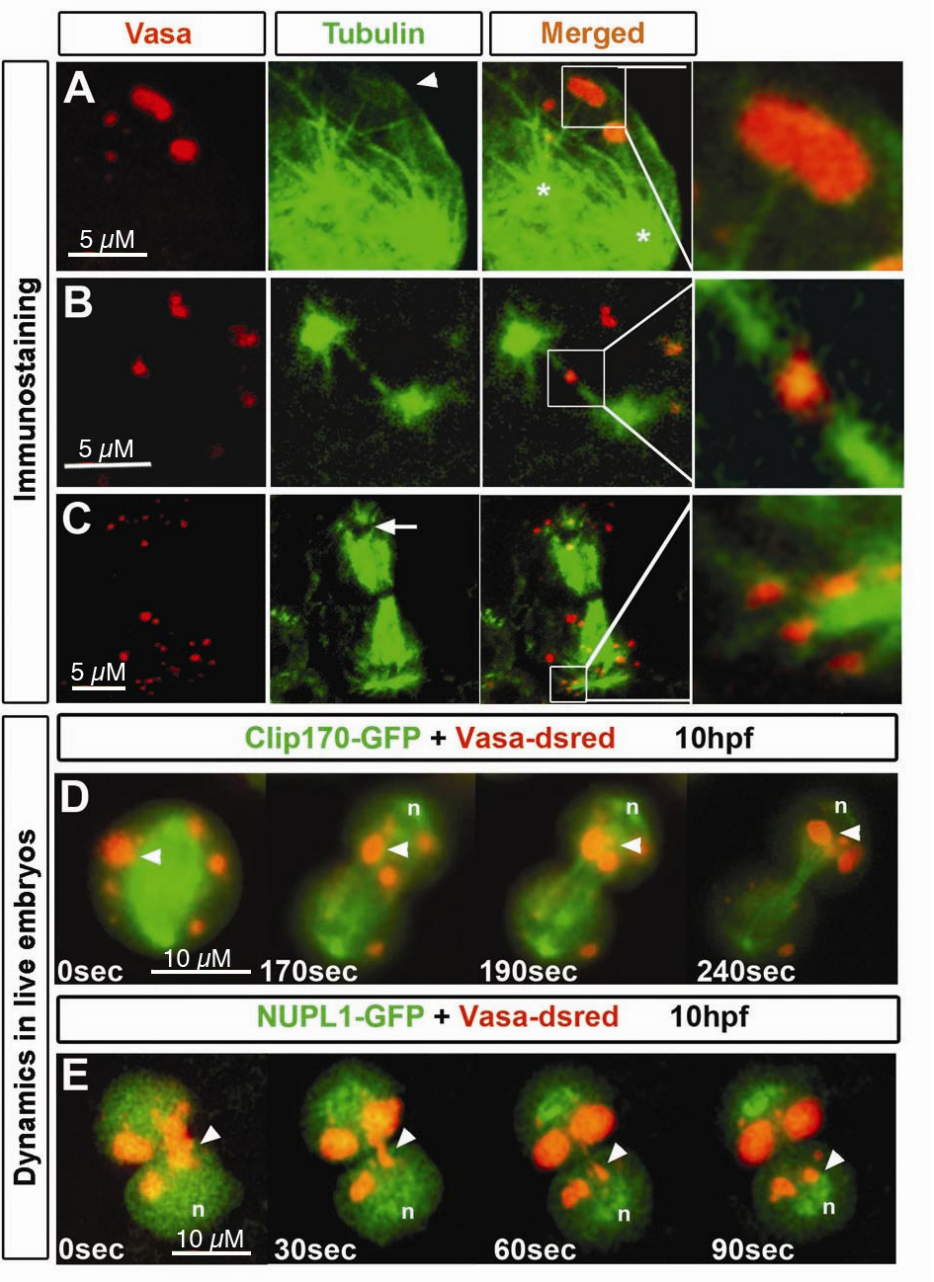
**Microtubules localization relative to germ cell granules during different stages of germ cell mitosis**. Immunostaining of germ cells undergoing cell division. Granules are labeled with anti-Vasa antibody and microtubules with anti-α-Tubulin antibody. A) During beginning of mitosis, α-Tubulin- expressing granules (arrowhead) appear connected to microtubular fibers (detail) projecting from what appear as centrosomes (asterisks). B) At a later stage of mitosis, when the two presumed centrosomes are located at the poles of the cell, projecting spindle microtubules reach at Vasa and α-Tubulin expressing granules (detail). C) When mitosis is close to termination, granules are enriched around the area where the nucleus reassembles (arrows). Specific colocalization of granules with astral microtubules is shown in detail. D) Germ cell granules (arrowhead) move towards the forming nucleus (n) through a path of spindle microtubules. E) Granules move to the area where the nucleus forms. Nuclear envelope reassembly was visualized by NUPL1. Elongated granules (arrowhead) move from the cytokinetic region to the site of the forming nucleus (n) and re-adopt a spherical shape upon arrival All experiments were performed on 10 hpf embryos.

Next, we sought to determine whether microtubules could be involved in granule segregation during mitosis. During cell division, microtubule organization and dynamics serve to ensure proper distribution of different cellular components such as chromosomes and the Golgi apparatus to the forming daughter cells [27,28]. We therefore followed the localization of granules of zebrafish germ cells during different stages of cell division with respect to microtubules. We observed that in germ cells stained for Vasa and α-Tubulin, some microtubular structures originating from the mitotic spindle appear to associate with germ cell granules during different stages of mitosis (Figure 5A,B,C arrowhead, arrow and details). When analyzing fixed material (Figure 5B), not all granules appear associated to microtubular structures. However, this is likely to result from microtubule disruption upon fixation and rather than absence of colocalization between these structures. In support of this notion, in live embryos germ cell granules move directionally and continuously along the spindle extension (Figure 5D arrowhead) towards the forming nucleus (Figure 5D, n depicts nucleus) during division (see Additional file 4 for movie). Granules elongate as they move from one cell to the other and re-adopt their spherical shape once they reach the area of the re-assembling nucleus (Figure 5E arrowheads). Functional experiments using microtubule-depolymerizing agents could not be performed, since upon disruption of the microtubular network, mitosis was arrested.

As previously mentioned, granules move towards the nuclear area after the termination of cell division. To examine the possibility that contraction of the cytokinetic ring might be the driving force for relocalization of germ cell granules to the nucleus, we inhibited cytokinesis by germ cell-specific expression of dominant negative RhoAN^19^. These cells are unable to form a contractile actin ring during cytokinesis due to the lack of functional RhoA, a protein known to regulate acto-myosin contraction [29,30]. As shown in Additional file 5, binucleated germ cells show normal localization of germ cell granules to the periphery of the nuclear envelop at the end of mitosis. Therefore, it is unlikely that the cytokinetic ring is involved in the segregation of granules into daughter cells.

### Localization and distribution of Dynein with respect to germ cell granules

As germ cell granules are localized in close proximity to microtubules during interphase (Figure 3) and directionally move along microtubule tracks during mitosis (Figure 5), we tested whether microtubule-dependent molecular motors are involved in granule organization within the cell.

**Figure 6:**
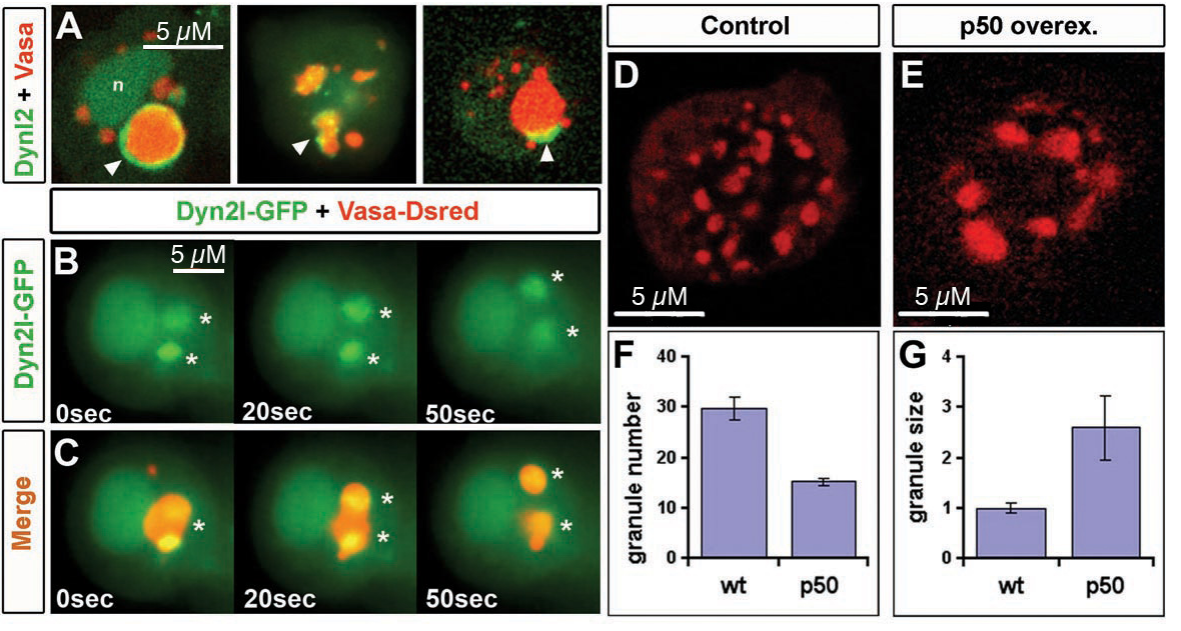
**Dynein localize to germ cell granules and is involved in granule fragmentation**. A) DynL2-GFP is localized to nucleus, cytoplasm and to germ cell granules. Granule DynL2-GFP localization patterns are diverse, ranging from ring-like structures around large granules (left panel, arrowhead, nucleus is depicted by n), dots within the granules (arrowhead, middle panel) or accumulations can also be observed on the periphery of such structures (arrowhead, right panel). Interphase cells where analyzed in 6 to 10 hpf embryos. Note that Vasa-dsRed and DynL2-GFP colocalization preferentially occurs in the largest granule of the cell. B) Germ cells of 9 hpf embryos expressing DynL2-GFP were analyzed *in vivo *by epifluorescence microscopy. DynL2-GFP is localized to a large germ cell granule where two centers of high concentration are observed preceding the division of the granule (asterisks in B,C). C) Merge of B together with Vasa-dsRed to visualize the granules. D) Immunostaining of control germ cells of 24 hpf embryos showing the normal number (shown graphically in F) and size (shown graphically in G) of Vasa expressing granules n = 33 cells and 191 granules (G). Dynein inhibition in germ cells of 24 hpf embryos by p50 overexpression reduces the number of granules per germ cell while the size of germ cell granules increases. n = 116 cells and 154 granules (E,G). TTtest for F: p < 1E-8. TTest for G: p < 1E-14.

To investigate the localization of the microtubule minus end-directed motor protein Dynein [31], we expressed a GFP fusion of the *dynein light chain *(*DynL2*) to label Dynein-containing complexes within primordial germ cells. DynL2-GFP is found in the nucleus and in the cytoplasm of the germ cells (Figure 6A,B), and significantly, it is also localized to germ cell granules (Figure 6A). Interestingly, DynL2-GFP shows a dynamic localization pattern in the granules, varying from ring-like structure (Figure 6A left panel), small dots within granules (Figure 6A middle panel), to asymmetric accumulations at the periphery of granules (Figure 6A right panel). Notably, DynL2-GFP is preferentially localized to larger granules (Figure 6A). The relevance of Dynein localization for granule organization is supported by findings such as those presented in Figure 6B and 6C where DynL2-GFP is localized to a large germ cell granule where two centers of high Dynein concentration are observed preceding the division of the granule. This observation is consistent with the idea that Dynein plays a role in the distribution of granule material within the cell during interphase. The observation that DynL2-GFP localizes preferentially to large granules (Figure 6A–C,) is in line with the hypothesis that Dynein is required for granule fragmentation.

### Dynein is necessary for proper germ cell granule distribution in germ cells

Considering the localization of Dynein to germ cell granules, we investigated the effect of Dynein inhibition on the distribution of germ cell granules. For this purpose, we inhibited Dynein function by overexpression of the Dynactin subunit Dynamitin (also referred to as p50) [32] in germ cells. Dynamitin overexpression is believed to disrupt the Dynactin complex, thereby inhibiting Dynein binding to its cargo [33]. Strikingly, in 24 hpf embryos, primordial germ cells in which Dynein function is inhibited show a strong reduction in the number of Vasa-positive granules (Figure 6E–F). Namely, whereas germ cells of wild type (wt) embryos exhibit an average of 30 ± 2.2 granules per cell, germ cells overexpressing Dynamitin contain on average only 15 ± 0.6 granules per cell (p < 0.001). Concomitant with the reduction in granule number, a dramatic increase in granule size was observed (Figure 6D,E,G). While the average cross section area of germ cell granules in control germ cells is 1.0 μm^2 ^(± 0.1) (n = 191 granules in 17 cells from 10 embryos), in cells overexpressing Dynamitin the average area is 2.1 μm^2 ^(± 0.2) (n = 154 granules in 26 cells from 10 embryos). These results indicate that Dynein is not involved in determining the total amount of germ cell granule material in the cell (which is roughly similar in control and experimental cells). Rather, dynein is involved in controlling the size of individual granules during the first 24 hours of zebrafish embryonic development. In addition to the effect on germ cell granules size and number, Dynamitin overexpression in germ cells resulted in a reduction in the number of germ cells to 78% (p < 0.001). We postulate that this phenotype reflects an additional role for Dynein in cell division.

### Tdrd7 plays a crucial role for structural integrity of granules in PGCs

We assumed that in addition to the microtubule network and the associated machinery, granule-specific components are likely to participate in regulating granules distribution, size and number in germ cells. We chose to focus on the zebrafish tudor-repeat-containing gene *Tdrd7*, whose transcription was found to be higher in germ cells as compared with somatic cells in a microarray screen. *Tdrd7 *belongs to a family of genes, some of which we found to be expressed in zebrafish germ cells (see Additional file 6). The transcript of Tdrd7 is localized to the germ plasm and is expressed in PGCs for at least the first days of development (see Additional file 6). In *Drosophila*, tudor function is important for germ cell specification and for the structural integrity of polar granules [34,35].

**Figure 7:**
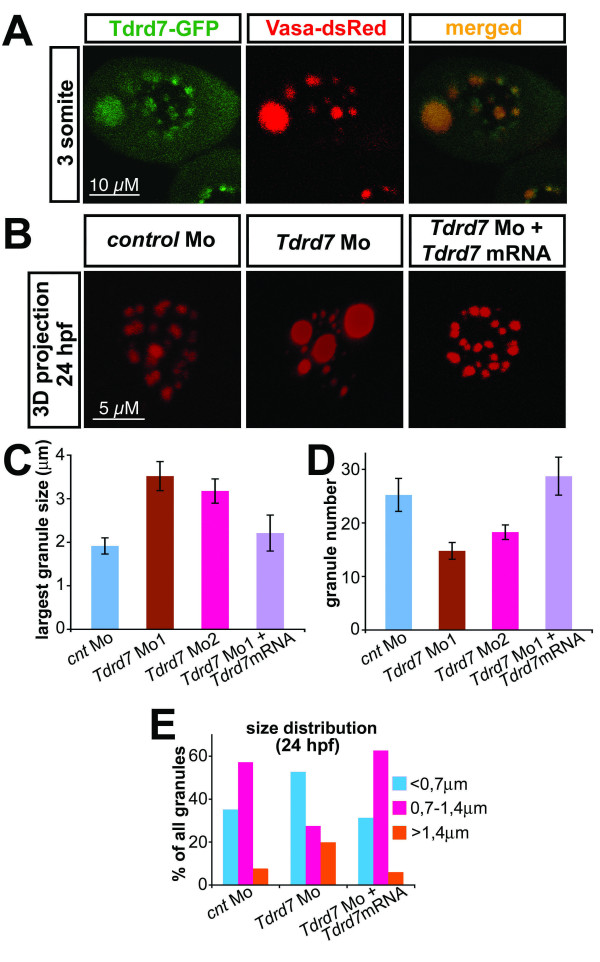
**Tdrd7 function is required for proper germ cell granule architecture**. A) Tdrd7-GFP fusion protein colocalizes with the germ cell granule marker Vasa-dsRed. A 3-somite stage embryo corresponds to 11 hpf embryo. B) Tdrd7 function is required the formation of uniform normal sized granules. A 3D projection of granules labeled with Vasa antibody in 24 hours old control cells (left panel), in Tdrd7 depleted cells (middle) and in cells from embryos co-injected with the antisense oligonucleotide directed against Tdrd7 and an RNA encoding Tdrd7 that is resistant for the inhibition by the antisense oligonucleotide (right). C) Size of the largest granule in 24 hpf PGC. N = 18 cells for control, 16 for morpholino 1 injected embryos (T-test compared with the control, p < 10^-3^), 19 for morpholino 2 and 16 cells in which the phenotype was reversed by co-injection of the morpholino resistant *Tdrd7 *RNA. D) Number of granules in 24 hpf PGC. N = 13 cells for control, 19 cells for morpholino 1 (T-test compared with the control, p < 10^-2^), 26 cells for morpholino 2 and 13 cells in which the phenotype was reversed by co-injection of the morpholino resistant *Tdrd7 *RNA., TTest p < 0,003). E) Granule-size distribution is increased in cells knocked down for Tdrd7 as the proportion of the very small and very large granules is increased. N = 223 granules for control, 150 granules for morpholino 1 and 132 granules in which the phenotype was reversed by co-injection of the morpholino resistant *Tdrd7 *RNA.

To examine Tdrd7 function, we initially determined the subcellular localization of the protein by expressing a Tdrd7-GFP fusion protein in germ cells. The Tdrd7 fusion is found in zebrafish germ cell granules, where it colocalizes with the germ cell granule protein marker Vasa-dsRed (Figure 7A). The localization of *Tdrd7 *was verified by 5' and 3' fusions, as well as by fusions to HA- and flag-tags. In addition to the nanos-3'UTR, it was fused to its own UTR yielding the same results (data not shown).

To determine whether Tdrd7 participates in controlling granule distribution, size or number, we used antisense morpholino oligonucleotides to inhibit *Tdrd7 *translation. No effect on PGC specification, division or migration upon inhibition of *Tdrd7 *translation was observed, as the number of PGCs in control embryos and Tdrd7 knock down embryos was equal at 20 hpf and the PGCs migrated properly to the position where the gonad develops (data not shown). Moreover, early PGC markers such as vasa, nanos, dead end, h1m, as well as the expression of the differentiation marker ziwi [18,36] were normally expressed in PGCs of Tdrd7 knock down embryos (see Additional file 7). Consequntly, embryos injected with Tdrd7 morpholino developed into fertile males and females (data not shown). However, a prominent phenotype of abnormal germ cell granule morphology was observed in 24 hpf treated embryos (Figure 7B middle panel). In these embryos we observed large granules that resembled those observed in early developmental stages in wild-type embryos (Figure 1D). In PGCs lacking Tdrd7, the average size of the largest granule in a cell is significantly increased (from 1,9 μm to 3,5 μm, Fig. 7C,E). In addition, we observed an increase in the number of small granules (Figure 7E), which is reflected by an alteration of granule size distribution in favor to extreme sizes (Figure 7E). This phenotype was readily reverted by co-injection of a morpholino-resistant mRNA encoding Tdrd7, confirming the specificity of the antisense oligonucleotide-induced phenotype (Figure 7B right panel). Thus, Tdrd7 function is important to guarantee the development of homogenous medium size granules over time and for normal number of granules in the germ cells. This phenotype was also demonstrated using Granulito as a marker for germ cell granules (see Additional file 8A,B). Tdrd7 function appears be required in maintaining germ cell granule integrity independently of microtubules and dynein function as the distribution of microtubules and dynein is not affected in Tdrd7 knock down PGCs (see Additional file 8C,D).

## Discussion

In this study we have identified a novel germ cell component *granulito*. The mRNA of this gene is maternally provided and becomes localized to the germ plasm, while the protein fusion localizes to zebrafish germ cell granules. Knock down experiments using *granulito *morpholino antisense oligonucleotides failed to induce a discernible phenotype. This suggests that the function of the protein might be either masked by a redundant protein(s), or that *granulito *function is not essential during early development. Alternatively, maternally-provided Granulito protein could be sufficient for carrying out the role at the stages tested.

Nevertheless, we have taken advantage of the newly identified gene and generated *granulito-dsRedEx *transgenenic fish to serve as a valuable tool for investigating germ cell granule distribution during very early stages of PGC development, stages that previously had been inaccessible for *in vivo *analysis in live embryos. We found that the morphology of germ-cell granules is transformed from large aggregates seen at early developmental stages into small granules that assume perinuclear localization in the cell. Such a transition is believed to allow the symmetrical distribution of the material to both daughter cells during cell division, thereby enabling an increase in germ cell number as the cells proliferate [37].

Following the distribution of germ-cell granules during the first 24 hours of embryonic development, we observed that as PGCs proliferate, the variation in granule sizes decreases. Analyzing the segregation of granules among daughter cells, we provide evidence that this process is not a random but rather follows an ordered segregation. Segregation of other cellular components (e.g. chromosomes [38,39] and the Golgi apparatus [28]) also follows an ordered partition and is dependent on microtubule function. Studies in *C.elegans *and zebrafish showed that microtubules are required for proper localization of germ plasm and germ cell specification [6,7,40]. Here we extended these studies to stages following PGC specification and demonstrate the presence of α-Tubulin within the granules and association of microtubules with granular structures. Furthermore, we show that during mitosis granules are found in close proximity to microtubules of the mitotic spindle, consistent with a role for microtubules in the ordered segregation of granules during cell division.

Granules are associated with microtubules during all stages of the cell cycle and seem to move along microtubule tracks during mitosis suggesting a functional interaction. We propose that microtubules of the spindle apparatus are part of a mechanism that ensures inheritance of granules by daughter cells after cell division. In this model, microtubules encounter and bind granules during centrosome repositioning in early stages of mitosis when these structures migrate towards the poles. Indeed, we could detect such an interaction during early germ-cell mitosis when presumed centrosomes are divided and move towards the poles (Figure 5A). Attachment of the granules to microtubules upon disassembly of the nuclear envelope and movement towards the forming poles by spindle microtubule pull, would allow a controlled segregation of these structures during the subsequent steps of mitosis.

Although we could not functionally address the role of microtubules for granule segregation during cell division due to their requirement for cell division *per se *[41], exposure to the microtubule inhibitor nocodazole demonstrated the importance of intact microtubule organization for the structural properties of germ cell granules (Figure 4). The effect on granule morphology is unlikely to represent an indirect effect of the drug on nuclear envelope structure, as nuclear envelope disassembly alone during mitosis does not affect granule structure.

The notion that microtubules are important for the morphology and translocation of the granules is further supported by the finding that the motor protein Dynein localizes specifically to germ cell granules. Moreover, inhibition of Dynein cargo-loading function by inhibiting Dynein-Dynactin interaction resulted in an increase in granule size and a reduction in their number (Figure 6F,G). Dynein is thus implicated in the breaking up granules into smaller units during development. Interestingly, the novel *Xenopus *protein Germes, which is important for germ plasm morphology, germ cell survival and migration of these cells in *Xenopus*, localizes specifically to germ plasm and directly interacts with Dynein light chains [42]. The presence of germ plasm components that directly interact with Dynein motors in *Xenopus *is consistent with the role we propose it plays in granule morphology and distribution in zebrafish. It is therefore reasonable to speculate that Dynein interacts with components of germ cell granules in zebrafish PGCs and that granules use microtubules to slide on, to be pulled and to break apart. Another possible scenario of dynein-granule interaction would be the association between components of the dynactin complex like Arp1 with Actin through Spectrin [43,44]. Such possibility is supported by the finding that actin is abundant in germ cell granules (data not shown). As described in the results, inhibition of dynein by dynamitin over expression led to a mild (20%) reduction in the total number of germ cells, cells that undergo 2–4 cell divisions during the first 24 hours of development. If the number of cell divisions is important for the developmental age perceived by the cells, then inhibition of cell division would result in germ cells having the germ plasm morphology of PGCs at an earlier developmental stage thus contributing to the severity of the observed germ plasm morphology phenotype.

In order to identify genes that are specifically expressed in granules and are involved in granule structural maintenance, we analyzed proteins containing tudor-repeat domains that were previously shown to be involved in polar granule structural integrity in Drosophila [35] and mice nuage maintenance [45,46].

Similar to the *Drosophila tudor *[34], *Tdrd7 *is localized to the germ plasm yet, formation of germ cells in Tdrd7-depleted zebrafish embryos appears unaffected. As our experimental approach does not inhibit maternal protein function, we cannot exclude a role for Tdrd7 protein in germ cell specification during early stages of zebrafish development.

Despite normal PGC specification and migration observed in *Tdrd7 *morphants, germ cell granule structure integrity is abnormal. This finding is in agreement with the findings of Arkov *et al*. that proposed that Tudor-domains serve as a docking platform for polar granule assembly in *Drosophila *and demonstrated that specific Tudor domains are required for proper granule architecture and germ cell formation [35]. In Drosophila embryos lacking Tudor function, polar granules fail to assemble and no PGCs are specified. Hypomorphic alleles show reduced number and size as well as abnormal morphology for polar granules, a phenotype that might signify a common function for the zebrafish and *Drosophila *proteins. As granule size at 4 hpf wt PGCs appeared larger than that in Tdrd7 knock-down cells at 24 hpf (compare Figure 1E*4 hpf wt *with 7E *24 hpf Tdrd7 knock down*), we assume that the observed phenotype is primarily caused by defects in the maturation process, although we could not exclude some Tdrd7 function required for keeping small granules separated.

The severe disruption of granule architecture due to Tdrd7 protein loss-of-function in early stages may hint at a critical role of the protein during later stages of germ cell development. For example, germ cells of mice deficient in Tdrd1 initially migrate and develop normally. However, male mice are sterile due to lack of mature sperm [45]. In these mutants, the inter-mitochondrial cement, an essential subset of nuage in mouse, is strongly reduced. Mice carrying mutant forms of RNF17, another Tudor domain containing protein, show a similar phenotype to that of Tdrd1 knockouts [46]. RNF17 localizes to a new form of nuage, RNF17-granules; RNF17 knockouts lack these granules and the male mice are infertile as well. As embryos injected with Tdrd7 morpholino (that inhibits translation only transiently) gave rise to fertile males and females we conclude that the early Tdrd7 function is not essential for fertility.

Since several genes contain tudor domains, *Tdrd7 *could be redundant with another yet-to-be-identified gene of the same family. As presented in the supplemental figure 3, we sought to identify other tudor domain containing proteins that are expressed in zebrafish germ cells. Indeed, we found that following the second day of development, *Tdrd1 *and *RNF17 *are specifically expressed in germ cells along with *Tdrd7*, suggesting a possible role for these genes in zebrafish germ cell development at later stages. Yet, Tdrd7 has been the only identified tudor domain-containing gene to be expressed during early zebrafish germ cell development. Interestingly, it has recently been shown that *Tdrd7 *mRNA in zebrafish is efficiently degraded in the soma by the same miRNA mechanism as for *nanos1 *mRNA while being protected from degradation in PGCs [47,48], lending further supports for the notion that Tdrd7 is important for germ cell development.

To elucidate the role of germ cell granules architecture in germ cell development, alteration of the function of genes described here during later stages of development will be required. For example, the identification of *Tdrd7 *zebrafish mutants or mouse knockouts that would allow the analysis of this effect during more mature stages of germ cell development. Similarly, germ cell-specific conditional knockout of Dynein may shed light on its role in later stages of germline development.

## Conclusion

Our study provides new insights into mechanisms responsible for proper germ cell granule architecture and inheritance during germ cell development. As similar structures are found in germ cells of different sexually reproducing animals, it would be interesting to examine whether the findings described here could be generalized to include a broad range of organisms.

## Methods

### Zebrafish strain and fish maintenance

Zebrafish (*Danio rerio*) of the AB genetic background were maintained, raised and staged as previously described [49,50].

### Identification of the granulito gene

The *granulito *(*gra*) cDNA (GenBank accession no. XM_685291) was identified in a screen for genes higher expressed in wild-type germ cells as compared to *dead end *knock down germ cells using the Affymetrix zebrafish genome array analysed with the dChip software [51]. The full-length cDNA was cloned into TOPOII (accession number EF643555). 5'RACE analysis confirmed that the cloned sequence contains the full-length of the 5'UTR.

### In situ hybridization

One-colour whole-mount in situ hybridization was performed as described previously [52] with modifications described elsewhere [53,54]. DIG-labeled antisense *granulito*-probe was synthesized using SP6 and DIG nucleotide mix (Roche) from a TOPOII plasmid containing *granulito*. The detailed cloning strategy of the gene is provided in the Additional file 9.

### Identification of Granulito orthologs

The Granulito protein sequence was blasted using tblastn on the NCBI homepage on EST database. The translation product of the *Homo sapiens *EST BU944391 and the *Xenopus leavis *EST CK799327 were used for alignment in ClustalW.

### Cloning of Tol2-kop-granulito-dsRedEx-nos1-3'UTR construct and generation of transgenic fish

The Tol2-*kop-granulito-dsRedEx-nos1*-3'UTR construct was generated as described in supplementary materials. For the analysis of germ cell granule structural changes during development, double transgenic fish were obtained by crossing positive females of the above described transgenic line with males carrying the Tol2-*kop-egfp-farnesyl-nos1*-3'UTR [55] transgene.

### RNA Expression Constructs

Capped sense RNA was synthesized with the mMessageMachine kit (Ambion) and microinjected into one-cell stage embryos. To direct protein expression to the PGCs, the corresponding open reading frames (ORFs) were fused upstream to the 3'UTR of the *nanos1 *(*nos1*-3'UTR) gene, facilitating translation and stabilization of the RNA in these cells [11]. To fluorescently label PGCs, pSP64T-*gfp-nos1*-3'UTR RNA was injected (210 pg per embryo) [11]. For studying the subcellular localization of Granulito protein, pSP64T-*granulito*-*eyfp-nos1*-3'UTR was used. For labeling the nuclear envelope in PGCs, pSP64T-*laminB2-mgfp-nos1*-3'UTR was used. To label germ cell granules we used the construct pSP64T-*vasa-dsRedEx-nos1*-3'UTR. For labeling zebrafish nuclear pore complexes the constructs pSP64T-*mgfp-NUPL1-nos1*-3'UTR and pSP64T-*NUP155-mgfp-nos1*-3'UTR were used. For labeling molecular motors, pSP64T-*egfp-kinesin11-nos1*-3'UTR and pSP64T-*Dyn2*-*egfp-nos1*-3'UTR were used. For disruption of Dynein-Dynactin function, zebrafish Dynamitin pSP64T-*dynamitin*-*nos1*-3'UTR was overexpressed. The constructs pSP64T-*H1M*-*egfp-nos1*-3'UTR, pSP64T-*egfp-farnesyl-nos1*-3'UTR, and pSP64T-*clip170-egfp-nos1*-3'UTR were used to label chromatin, plasma membrane and microtubules respectively. To inhibit cytokinesis, mRNA of pSP64T-*N19RhoA-nos1*-3'UTR was injected.

Full length *Tdrd7 *was cloned and confirmed with 5'Race (accession number EF643554). For studying the subcellular localization of Tdrd7 protein, pSP64-*egfp-Tdrd7-*3'UTR was used. The detailed cloning strategy is provided in the Additional file 9.

### Morpholino Knockdown Experiments and Drug treatment

Knock down experiments with *granulito *morpholino antisense oligonucleotide (0.15–3 pmol, CGTCCTCTGCCTCTGTCATTTTTAA, GeneTools) were performed. To examine the functionality of the morpholino, *vasa-dsRedEx-nos1-*3'UTR and *granulito-EYFP-nos1*-3'UTR (each 300 pg RNA) were co-injected with 0.3 pmol *granulito *morpholino into the yolk of one-cell stage embryos and specific inhibition of EYFP expression was observed. Knockdown experiments with *Tdrd7 *morpholino antisense oligonucleotide injection (0.3 pmol, GeneTools) were performed using two independent morpholinos Mo1 (AACCAACTCCACGTCACTCATCCTG) and Mo2 (TCCTGCCGTTTTCTCTTCACACTTG). To examine the function of the morpholinos, *vasa-dsRedEx-nos1-*3'UTR (300 pg RNA) and *Tdrd7-eyfp-nos1*-3'UTR (600 pg RNA) were co-injected with 0.3 pmol Tdrd7 morpholino into the yolk of one-cell stage embryos and a specific inhibition of EYFP was observed. In addition, the *Tdrd7 *morpholino induced phenotype was reverted by injection of a morpholino resistant *Tdrd7 *mRNA. For this, *Tdrd7 *was amplified with a forward primer containing mutations at the morpholino-binding site 5'TTTAGATCTACCATGAGCGATGTCGAATTAGTGAAGAAGATGCTGCGAGC3' and reverse primer 5'AAATCTAGATAATACAACAAAACCTGAACACC3' from ovary cDNA.

Nocodazole (CALBIOCHEM) was prepared as a stock solution of 10 mg/ml in DMSO, and used at a concentration of 1 μg/ml. Embryos were injected with mRNA generated from the constructs pSP64T-*vasa*-*dsRedEx*, pSP64T-*H1M-gfp-nos1-3'UTR*, and pSP64T-*LaminB2-gfp-nos1-3'UTR *and grown for 11 hours. *In vivo *epifluorescence microscopy movies where generated as the embryos where exposed to the drug. Frames were captured after 6 hours of exposure to the drug.

### Immunohistochemistry

For general immunostainings, embryos were fixed with 4% paraformaldehyde/PBS for 1 hour at room temperature. Alternatively, when labeling cytoskeleton structures, 100% methanol at -20°C was used in order to preserve structures. After fixation, the embryos were washed three times for 5 minutes with PBTX (PBT, 0.2% Triton ×-100) and subsequently blocked with PBTB (PBT, 0.2% Triton ×-100, 1% BSA) for 1 hour. The embryos were incubated in the blocking solution containing the primary antibody overnight at 4°C. anti-GFP antibody was obtained from Santa Cruz Biotechnology 1:200, anti-vasa antibody 1:2000 (Kindly provided by C. Nüsslein-Volhard [9]). The monoclonal antibody against α-tubulin (Sigma clone DM1A) was used at a 1:1000 dilution. Anti-nucleoporin monoclonal antibody (MAb414, Covance Research (Hiss Diagnostics) catalog number MMS-120R) was used at a 1:1000 dilution. The embryos were then washed with PBTX 8 times for at least 30 minutes each. Thereafter, they were incubated with the secondary antibody (1:200 Alexa Fluor488-conjugated anti-rabbit IgG and 1:200 Alexa Fluor546-conjugated anti-mouse IgG, Molecular Probes) overnight at 4°C. The embryos were then washed with PBTX for several hours and observed by confocal microscopy (Leica TCS SL).

### Fluorescence microscopy and imaging of live cells

Images were obtained using a Zeiss Axioplan2 microscope controlled by the Metamorph software (Universal Imaging). High magnification time-lapse movies were generated using a 63× objective capturing frames at 10-second intervals. For time-lapse analysis of early granule structure dynamics, eggs produced by *kop*-*granulito-DsRedex*-*nos*1-3'UTR transgenic females were used.

### Confocal imaging

Images were obtained using a Leica TCL SL microscope. For time lapse, frames were captured in 10 sec interval. Z-stacks were taken in the optimal spacing mode. 3-D reconstruction was performed using the ImageJ software (rsb.info.nih.gov/ij/). All images were taken using pinhole values that ranged from 1.28 to 1.84.

### Granule numbers and size measurements

Total number of granules was calculated using 3D reconstructions of confocal stack series of germ cells labeled for Vasa protein. 3D reconstructions were generated using the ImageJ software. Granule area calculations where done by measuring length and width of each granule using the internal Leica software. When fluorescent microscope images were used to calculate the size, number of pixels were measured in ImageJ and then calculated into mm considering camera resolution.

## Authors' contributions

MJS identified and characterized *granulito *and *Tdrd7*. NCM analyzed the role of the microtubular network and its motor protein in germ cell granule segregation and distribution. KD was involved in the initial screen, which led to the identification of *granulito *and *Tdrd7*. L-IN cloned the Dynein constructs. JS and ER participated in the design of the study. All authors read and approved the final manuscript.

## Supplementary Material

Additional file 1 ***granulito *morpholino inhibits translation of *granulito-yfp *mRNA**. *vasa-dsRed *and *granulito-yfp *mRNAs were coinjected with *control *morpholino (left panel) or *granulito *morpholino (right panel). While in the control both Vasa-dsRed and Granulito-YFP are visible, only Vasa-dsRed can be detected in *granulito *morphants. This demonstrates that the translation of *granulito *mRNA is efficiently and specifically blocked by the morpholino. Click here for file

Additional file 2 **Re-organization of granules around the nuclear envelop after cytokinesis**. At the end of cytokinesis granules move towards the forming nuclear envelope. Granule movement takes place after the nuclear envelope has reassembled as visualized by the Lamin B2 network. The plasma membrane is visualized in green by Farnesyl-GFP, granules in red by Vasa-dsRed and nucleus in green labeled with LaminB2-GFP in germ cells of 10 hpf embryos. Click here for file

Additional file 3 **Table of granule distribution among daughter cells**. Dividing PGCs of embryos from 10–12 hpf were followed in time laps movies. Germ cell granules were labeled with Vasa-dsRedEx. Click here for file

Additional file 4 **In vivo dynamics of germ cell granules and microtubules during germ cell division**. Granules move towards the forming nucleus along spindle microtubules. Some granules colocalize with astral microtubules. Microtubules are labeled with Clip170-GFP and granules with Vasa-dsRed in germ cells of 10 hpf embryos. Click here for file

Additional file 5 **Inhibition of cytokinesis does not interfere with perinuclear localization of granules following nuclear division**. Epifluorescence pictures of germ cells labeled with Farnesylated-GFP, H1M-GFP for visualizing plasma membrane, and Vasa-dsRed for germ cell granules. A) Cells expressing RhoAN19 do not undergo cytokinesis and polynucleated cells are observed. Although no cytokinetic ring is formed, granules reach the nuclear envelope (n) and readopt perinuclear localization (arrowhead) after division of the nucleaus. B) Interphase cells show normal perinuclear distribution of germ cell granules in both nuclei. Click here for file

Additional file 6 **Expression pattern of Tudor domain containing proteins in zebrafish embryos and larvas**. In situ hybridization for zebrafish homologs of previously described tudor domain containing genes *RNF17*, *Tdrd1*, *Tdrd5*, *Tdrd6 *and *Tdrd7 *at different stages starting from 4cell stage until 5 dpf. For *RNF17 *germ cell specific expression was observed starting from 24 hpf. For *Tdrd1 *germ cell specific expression was observed starting from 48 hpf. For *Tdrd5 *no expression could be observed in the analyzed stages. For *Tdrd6 *faint staining at the region where germ cells reside is visible at 3 dpf and 5 dpf. *Tdrd7 *expression is observed specifically at the cleavage furrow of the 4cell stage where the germ plasm is localized and continues to be expressed specifically in the germ cells throughout the analyzed stages. Click here for file

Additional file 7 **In situ hybridization of PGC markers in Tdrd7 morphants**. The early PGC markers nanos, vasa, dead end and h1m are normally expressed in PGCs of Tdrd7 morphants as judged by the comparison to control embryos. ziwi, a marker for PGC differentiation after the migratory stages starts its expression normally in Tdrd7 morphant germ cells. Click here for file

Additional file 8 **Verification of the Tdrd7 knock down phenotype**. A) 3D projection of granules labeled with Granulito-EYFP in 24 hours old control cells (left panel), in Tdrd7 depleted cells (right panel) verifies that Tdrd7 function is indeed required for the formation of uniform normal sized granules. B) Granulito and Vasa colocalize as well in the Tdrd7 morphant situation with each other. C) Coexpression of the GFP fusion to dynein light chain2 and Vasa DsRed in Tdrd7 morphant PGCs shows a comparable dynamic localization of Dynein in germ cell granules as it is known for wildtype PGCs. D) Using Clip170-GFP as a marker for microtubular networks shows normal network for interphase as well as mitotic PGCs. Click here for file

Additional file 9 Supplemental Methods. Click here for file
