# Supplementary figures and images for: Control over the morphology and segregation of Zebrafish germ cell granules during embryonic development
Source: BMC Dev Biol. 2008 May 28;8:58. doi: 10.1186/1471-213X-8-58 (PMC2441585; doi:10.1186/1471-213X-8-58)

*control* Mo

*granulito* Mo

Vasa-dsRed

10  $\mu$ M

Granulito-EYFP

merged

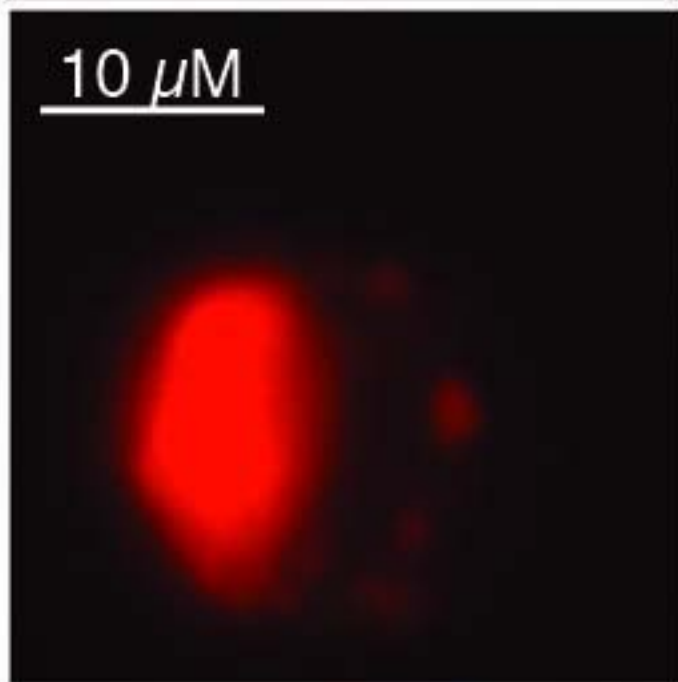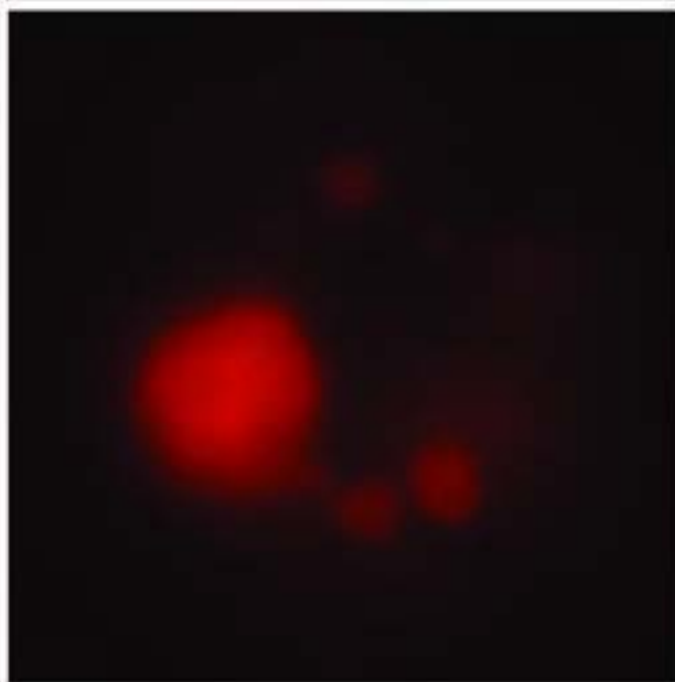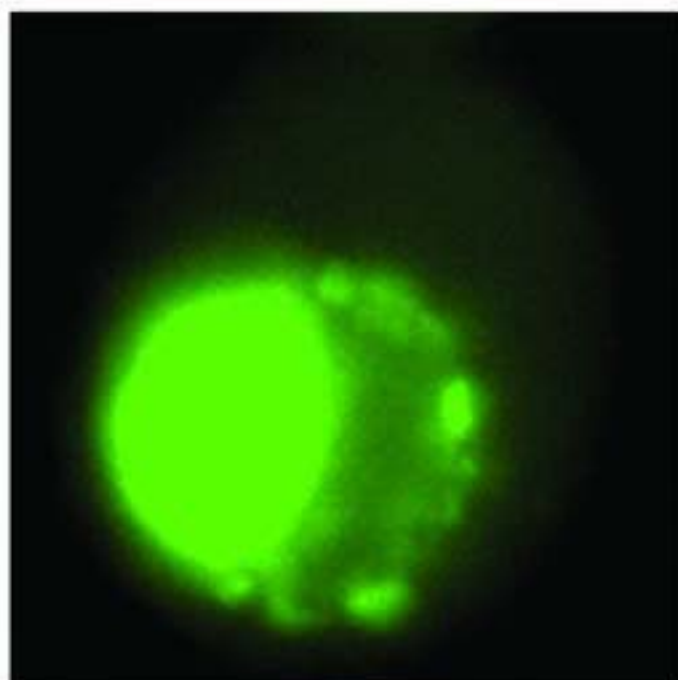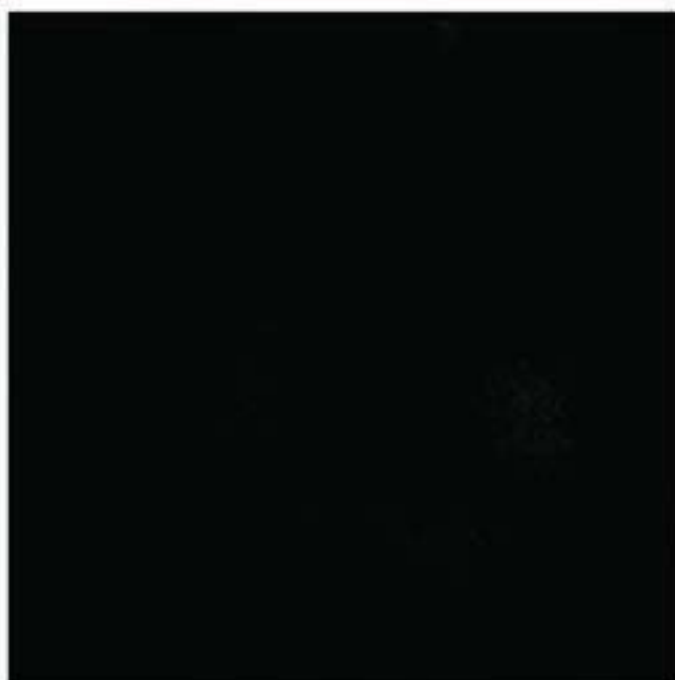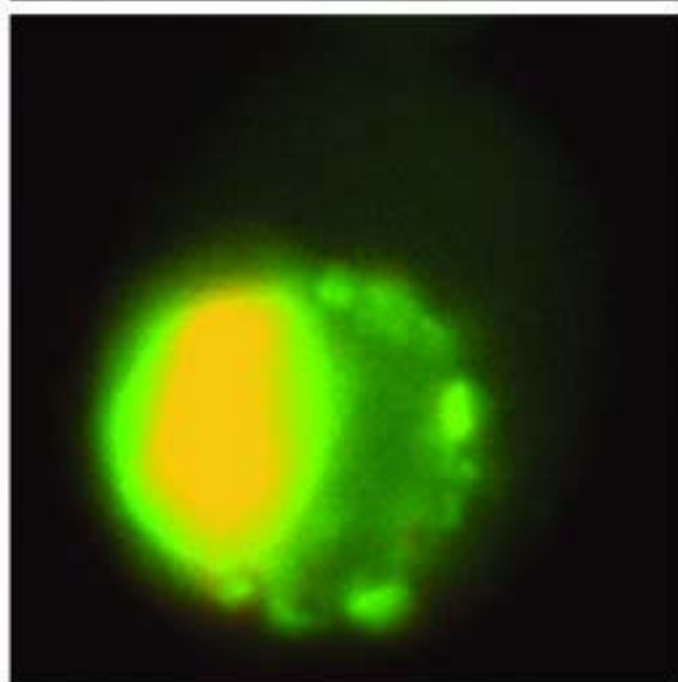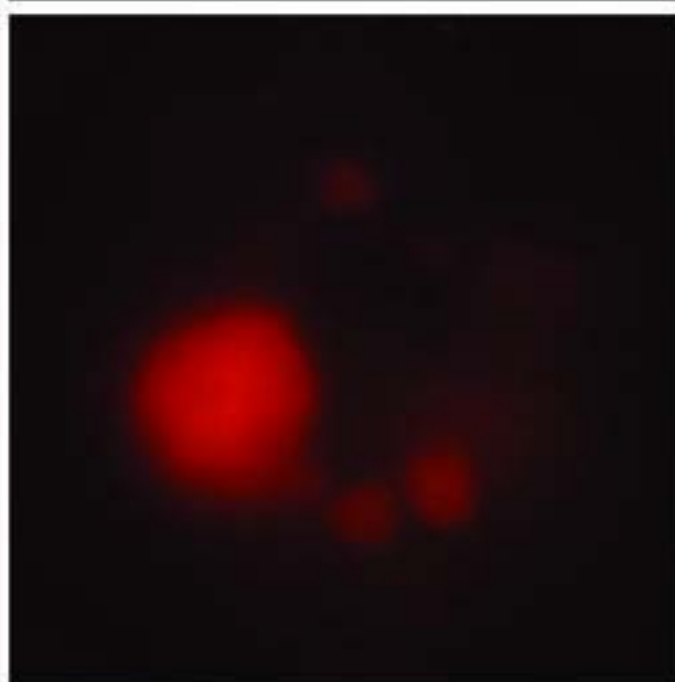

Supplement: Additional file 1 — granulito morpholino inhibits translation of granulito-yfp mRNA. vasa-dsRed and granulito-yfp mRNAs were coinjected with control morpholino (left panel) or granulito morpholino (right panel). While in the control both Vasa-dsRed and Granulito-YFP are visible, only Vasa-dsRed can be detected in granulito morphants. This demonstrates that the translation of granulito mRNA is efficiently and specifically blocked by the morpholino. [file 1471-213X-8-58-S1.pdf]

**End of mitosis**

**Interphase**

**Nucleus-Vasa**

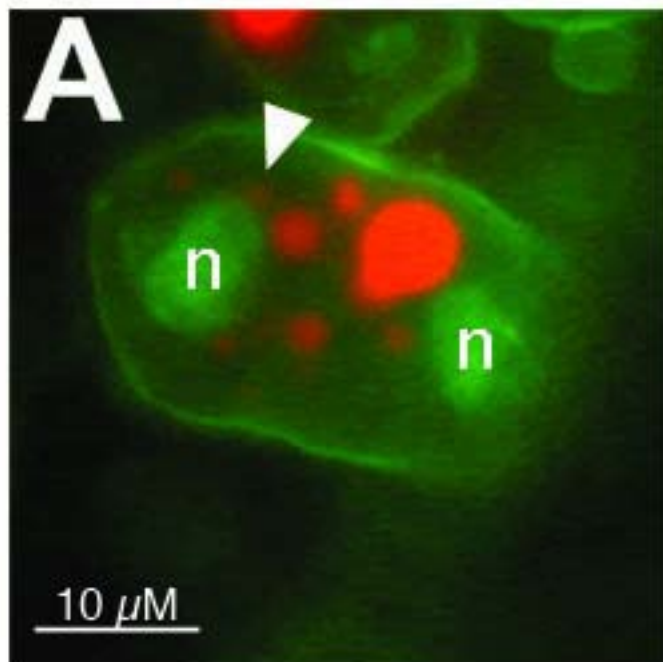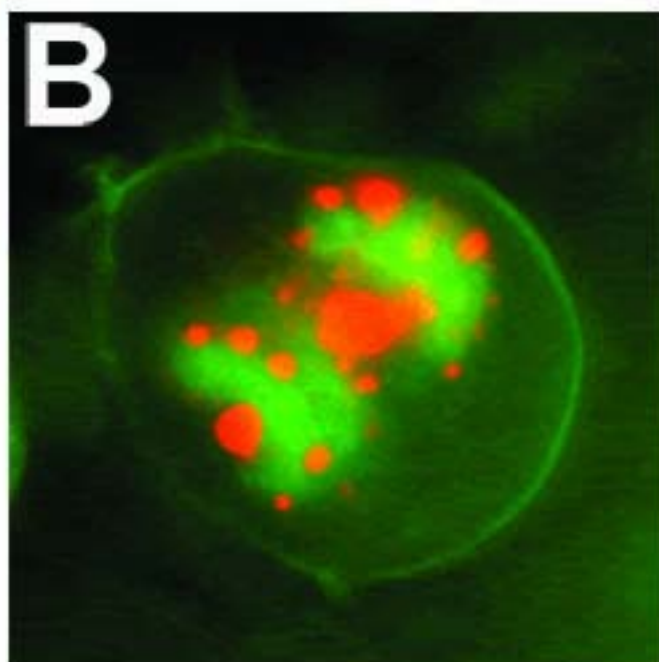

Supplement: Additional file 5 — Inhibition of cytokinesis does not interfere with perinuclear localization of granules following nuclear division. Epifluorescence pictures of germ cells labeled with Farnesylated-GFP, H1M-GFP for visualizing plasma membrane, and Vasa-dsRed for germ cell granules. A) Cells expressing RhoAN19 do not undergo cytokinesis and polynucleated cells are observed. Although no cytokinetic ring is formed, granules reach the nuclear envelope (n) and readopt perinuclear localization (arrowhead) after division of the nucleaus. B) Interphase cells show normal perinuclear distribution of germ cell granules in both nuclei. [file 1471-213X-8-58-S5.pdf]

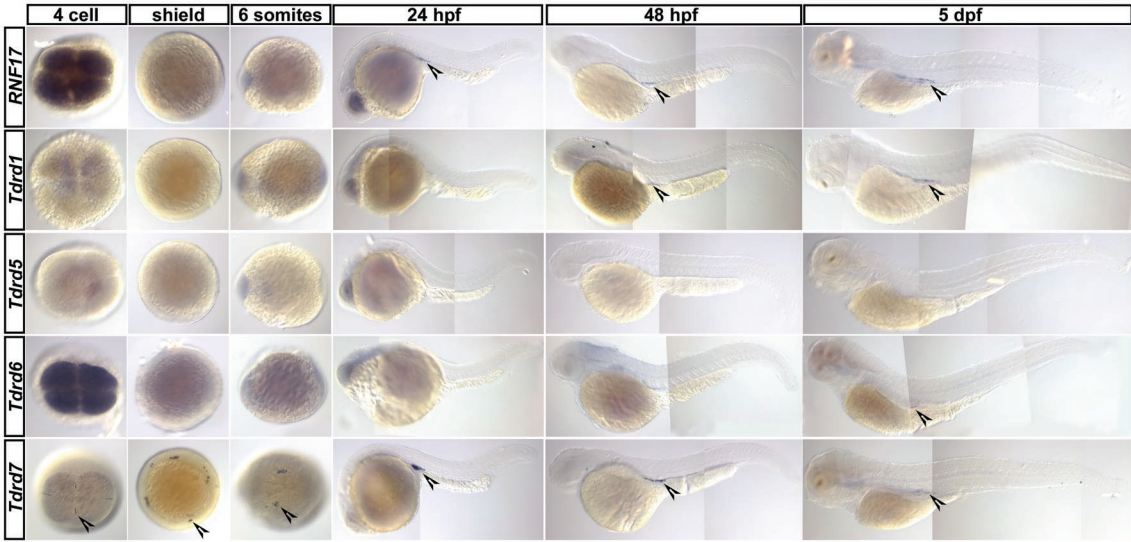

Supplement: Additional file 6 — Expression pattern of Tudor domain containing proteins in zebrafish embryos and larvas. In situ hybridization for zebrafish homologs of previously described tudor domain containing genes RNF17, Tdrd1, Tdrd5, Tdrd6 and Tdrd7 at different stages starting from 4cell stage until 5 dpf. For RNF17 germ cell specific expression was observed starting from 24 hpf. For Tdrd1 germ cell specific expression was observed starting from 48 hpf. For Tdrd5 no expression could be observed in the analyzed stages. For Tdrd6 faint staining at the region where germ cells reside is visible at 3 dpf and 5 dpf. Tdrd7 expression is observed specifically at the cleavage furrow of the 4cell stage where the germ plasm is localized and continues to be expressed specifically in the germ cells throughout the analyzed stages. [file 1471-213X-8-58-S6.pdf]

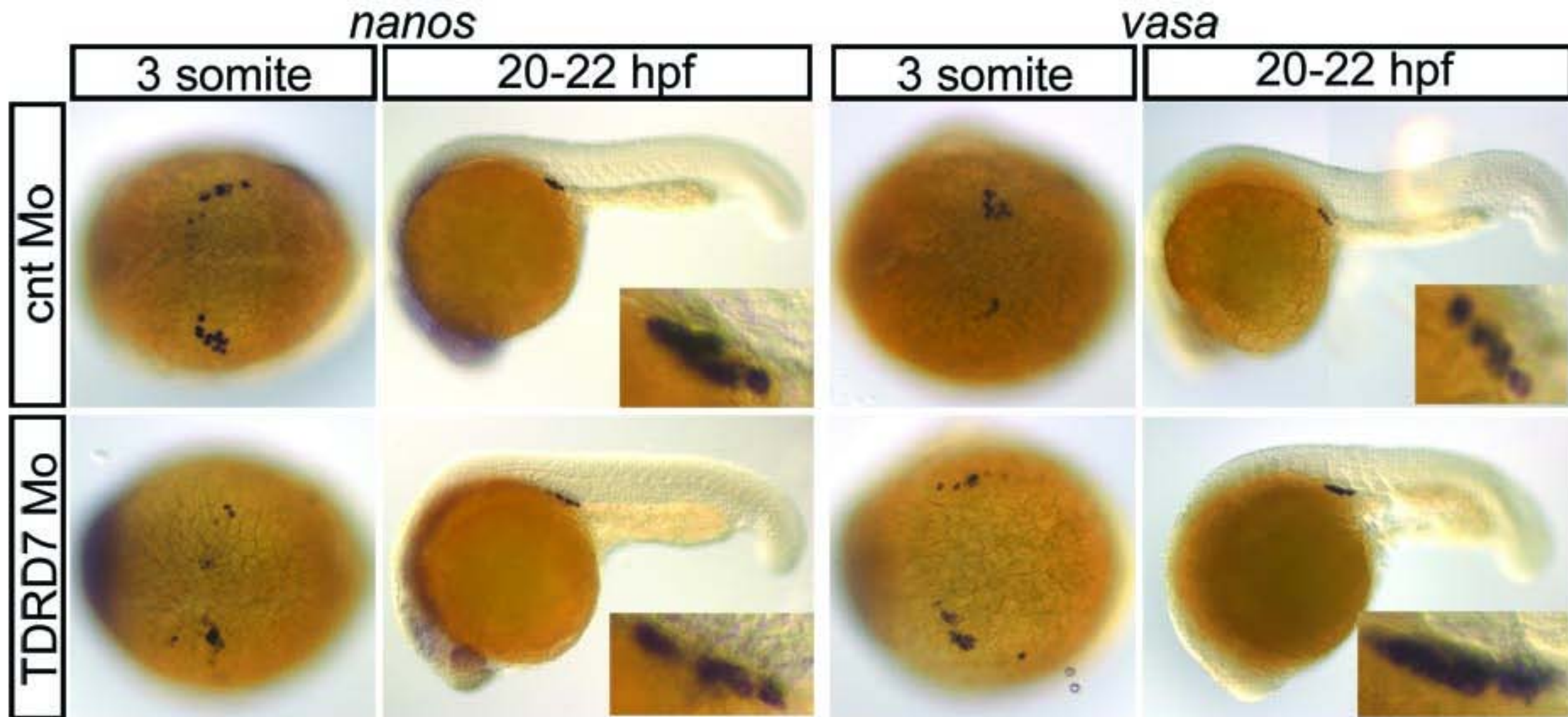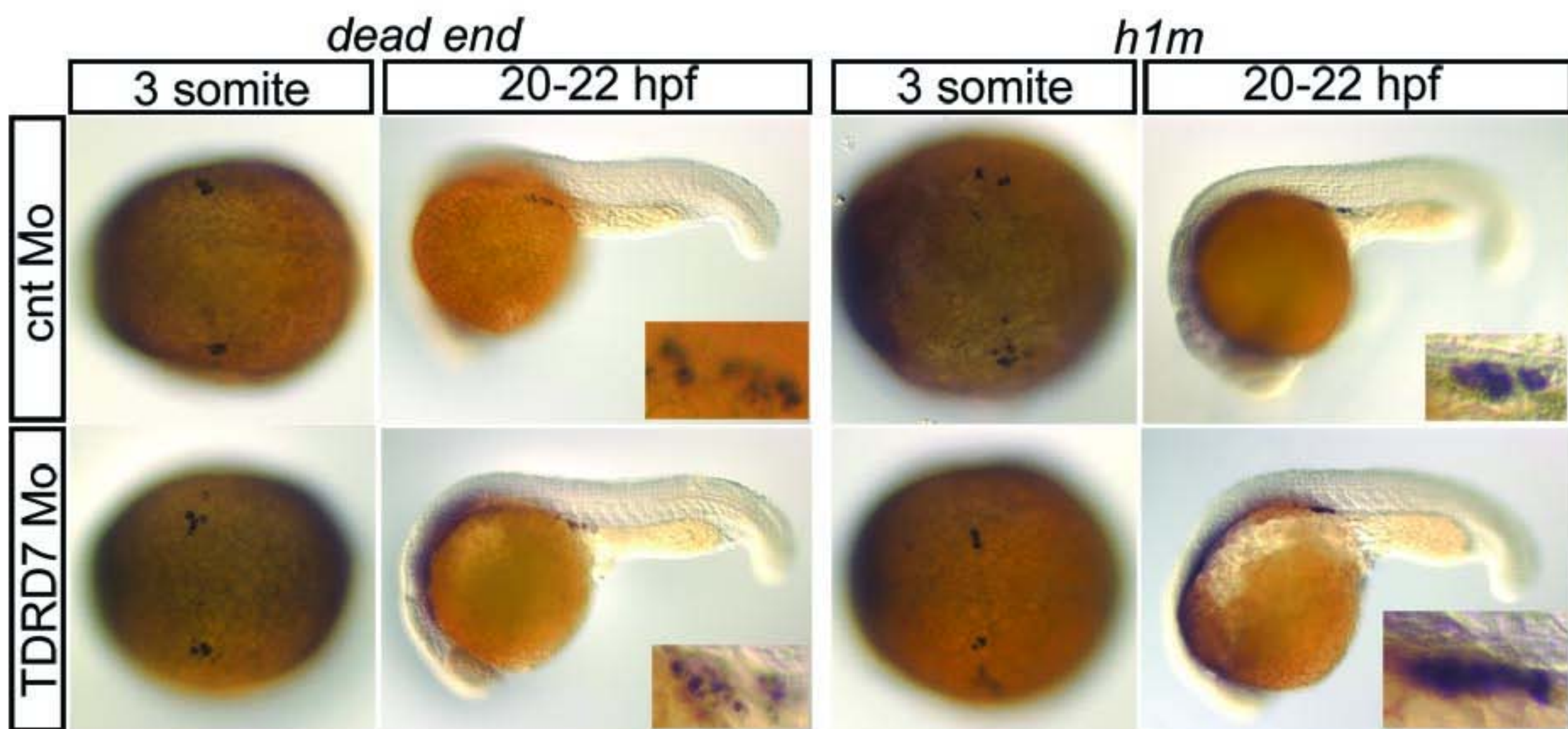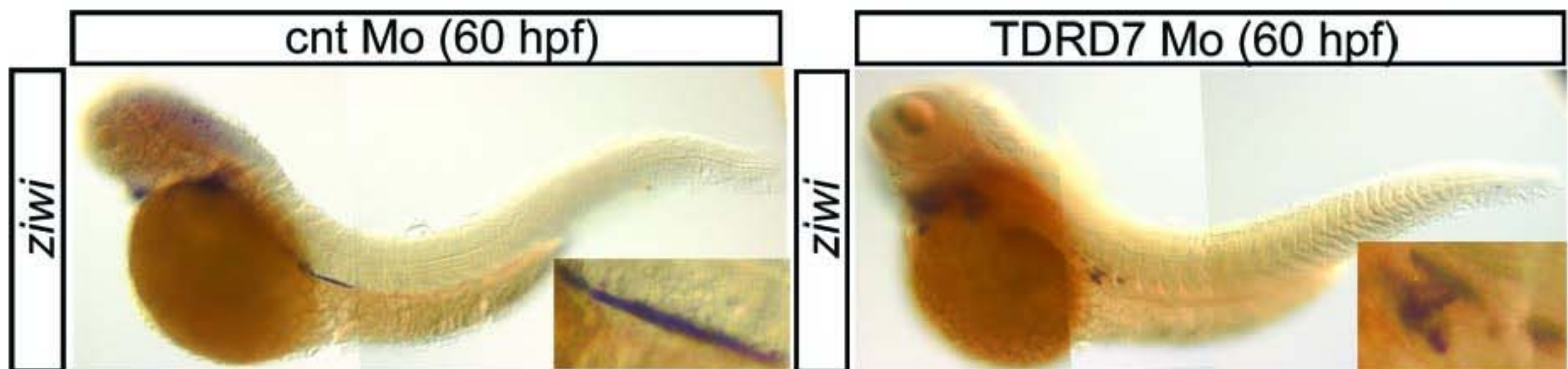

Supplement: Additional file 7 — In situ hybridization of PGC markers in Tdrd7 morphants. The early PGC markers nanos, vasa, dead end and h1m are normally expressed in PGCs of Tdrd7 morphants as judged by the comparison to control embryos. ziwi, a marker for PGC differentiation after the migratory stages starts its expression normally in Tdrd7 morphant germ cells. [file 1471-213X-8-58-S7.pdf]

**A**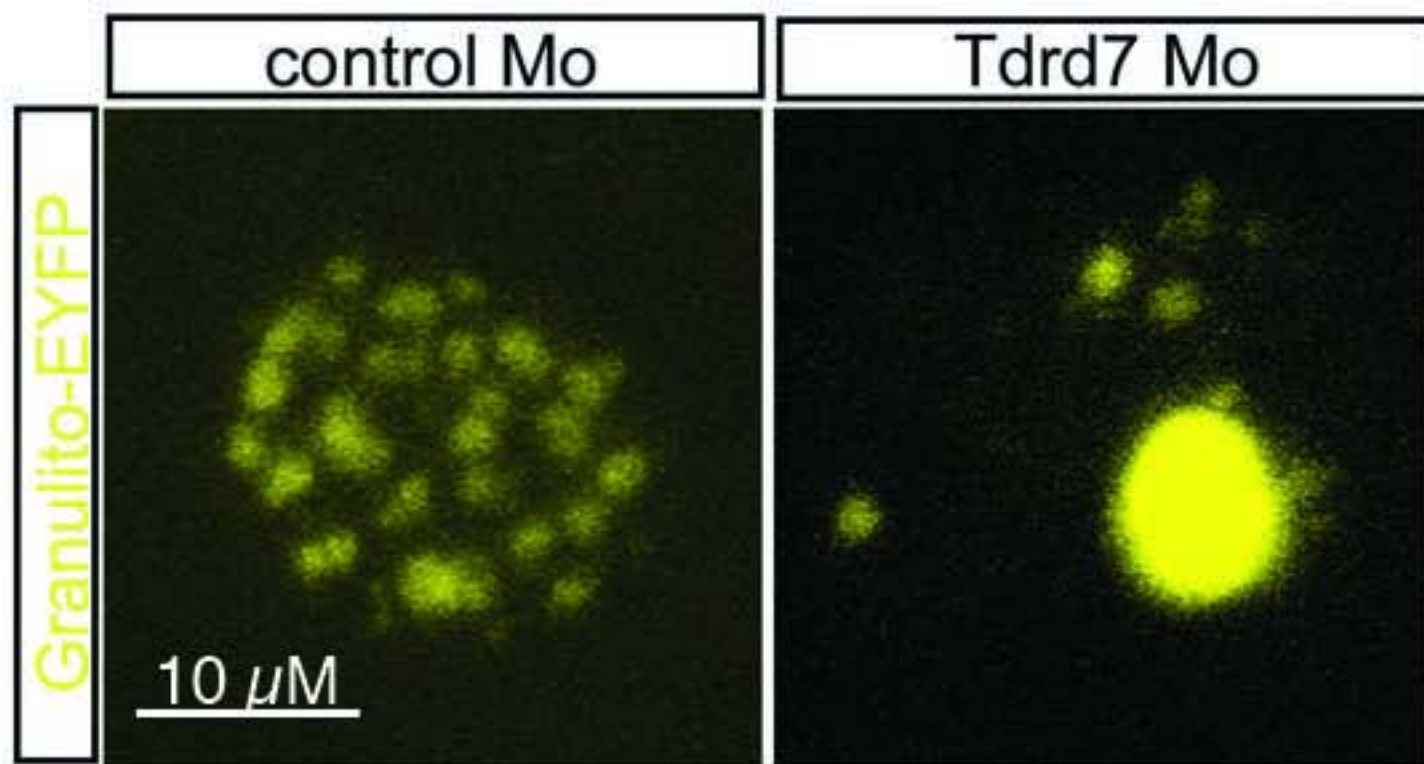**B**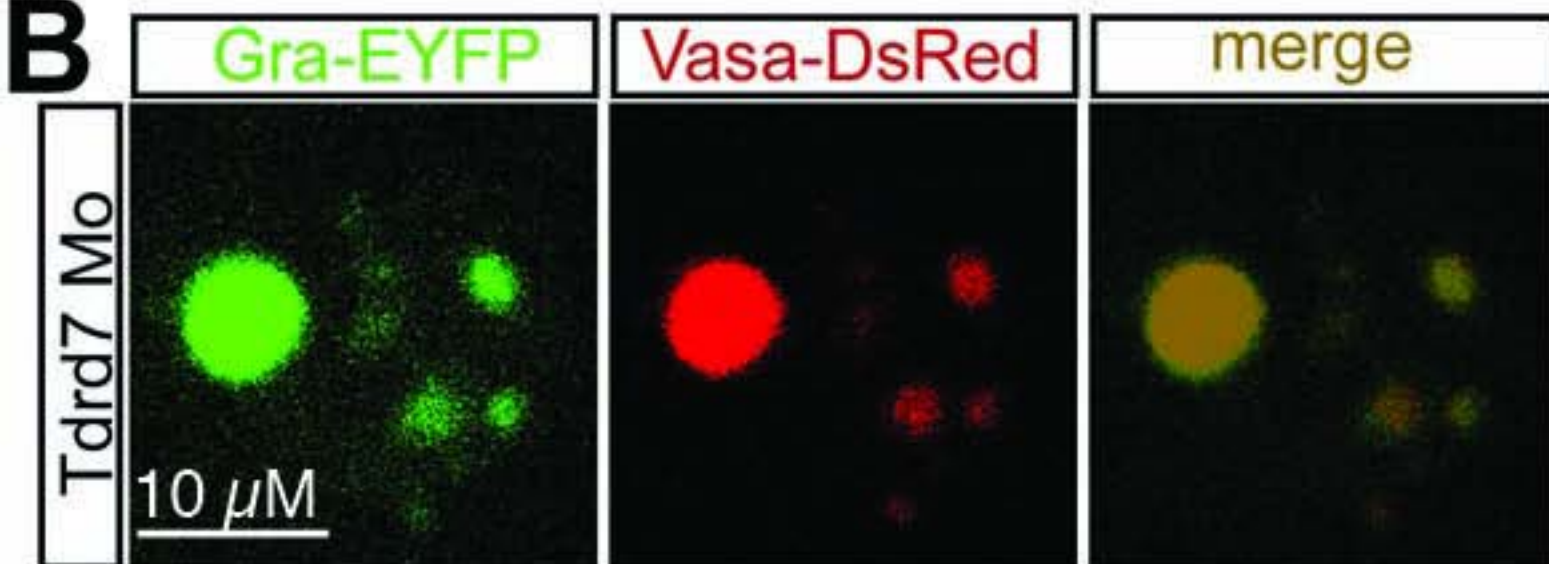**C**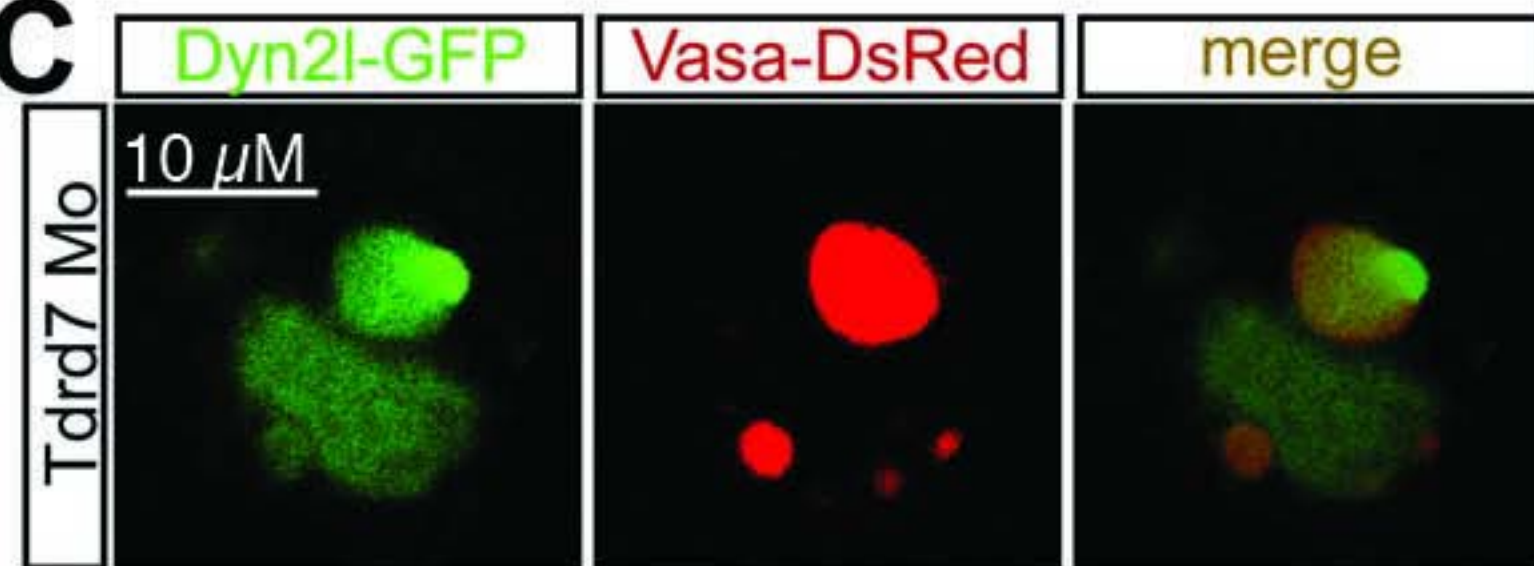**D**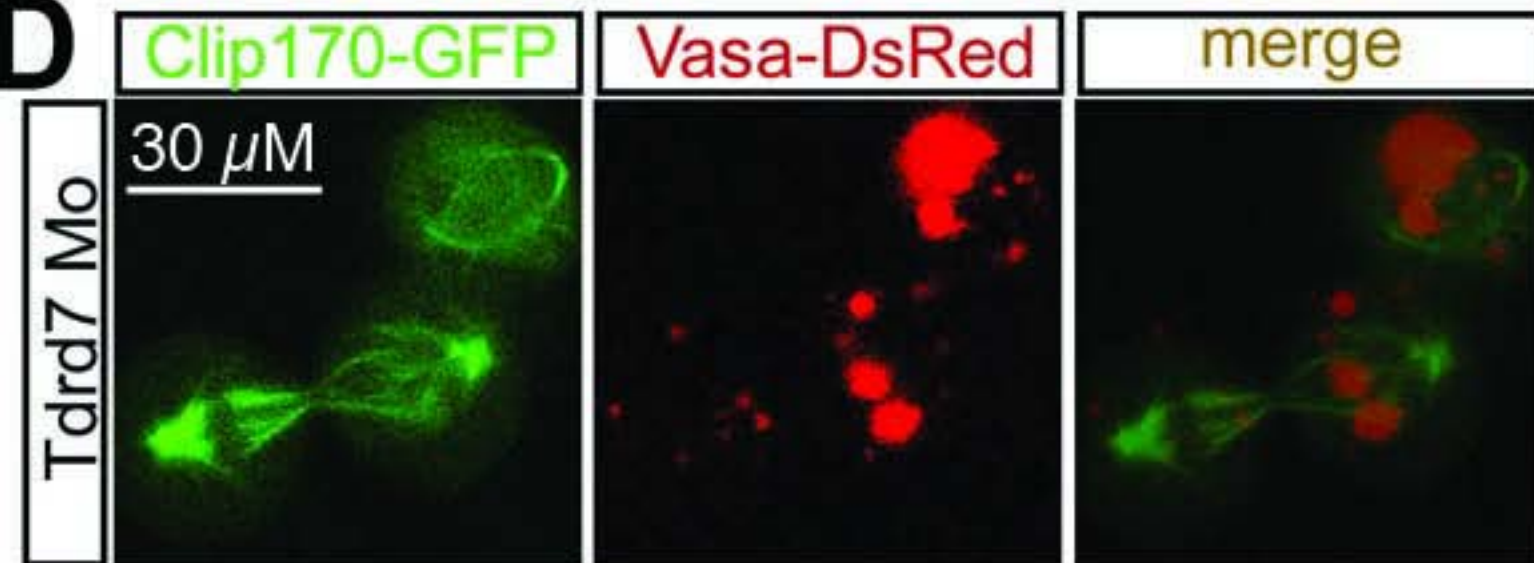

Supplement: Additional file 8 — Verification of the Tdrd7 knock down phenotype. A) 3D projection of granules labeled with Granulito-EYFP in 24 hours old control cells (left panel), in Tdrd7 depleted cells (right panel) verifies that Tdrd7 function is indeed required for the formation of uniform normal sized granules. B) Granulito and Vasa colocalize as well in the Tdrd7 morphant situation with each other. C) Coexpression of the GFP fusion to dynein light chain2 and Vasa DsRed in Tdrd7 morphant PGCs shows a comparable dynamic localization of Dynein in germ cell granules as it is known for wildtype PGCs. D) Using Clip170-GFP as a marker for microtubular networks shows normal network for interphase as well as mitotic PGCs. [file 1471-213X-8-58-S8.pdf]
