# Supplementary material for: Control over the morphology and segregation of Zebrafish germ cell granules during embryonic development
Source: BMC Dev Biol. 2008 May 28;8:58. doi: 10.1186/1471-213X-8-58 (PMC2441585; doi:10.1186/1471-213X-8-58)
Supplement: Additional file 3 — Table of granule distribution among daughter cells. Dividing PGCs of embryos from 10–12 hpf were followed in time laps movies. Germ cell granules were labeled with Vasa-dsRedEx. [file 1471-213X-8-58-S3.doc]

Table 1 Granule distribution among daughter cells. Dividing PGCs of embryos from 10-12 hpf were followed in time laps movies. Germ cell granules were labeled with Vasa-dsRedEx.

|  | Total granules | daughter 1 | daughter 2 |
| --- | --- | --- | --- |
|  | 21 | 11 | 10 |
|  | 20 | 10 | 10 |
|  | 21 | 11 | 10 |
|  | 13 | 7 | 6 |
|  | 25 | 13 | 12 |
|  | 18 | 9 | 9 |
|  | 17 | 9 | 8 |
|  | 17 | 9 | 8 |
|  | 16 | 8 | 8 |
|  | 23 | 12 | 11 |
|  | 17 | 9 | 8 |
|  | 18 | 9 | 9 |
|  | 14 | 7 | 7 |
|  |  |  |  |
| average | 18,5 | 9,5 | 8,9 |
