# Supplementary material for: Control over the morphology and segregation of Zebrafish germ cell granules during embryonic development
Source: BMC Dev Biol. 2008 May 28;8:58. doi: 10.1186/1471-213X-8-58 (PMC2441585; doi:10.1186/1471-213X-8-58)
Supplement: Additional file 9 — Supplemental Methods. [file 1471-213X-8-58-S9.doc]

**Supplemental Methods**

***Cloning description and primers.***

*Granulito* was amplified from maternal cDNA using primers 5’AAAAATGACAGAGGCAGAGGA3’ and 5’AAGTGACAGTTCTATTTTGCTTGC3’ and cloned into TOPOII (Invitrogen).

The Tol2-*kop-granulito-dsRedEx-nos1*-3’UTR construct was generated by replacing *egfp-farnesyl* with *dsRedEx* in the Tol2-*kop-egfp-farnesyl-nos1*-3’UTR [1] and inserting the PCR amplification product of *granulito* (using the forward primer 5’AAACCATGGATGACAGAGGCAGAGGACG3’ and the reverse primer 5’GGGTCAGCTTGCCGTAGGTG3’ in frame upstream of *dsRedEx*. The purified plasmid DNA was coinjected with sense RNA encoding the Tol2 transposase into one-cell stage zebrafish embryos [2]. The transgene directs Granulito-dsRedEx expression to PGCs, labeling germ cell granules.

*granulito* was amplified from cDNA (a mixture of ovary + mid-somitogenesis stages) with forward primer (TTTAGATCTGTTTAAAAATGACAGAGGCAG) and reverse primer (TTTGGATCCAAGTCAGACAAGTGTTTGTAGT) cloned in frame upstream of *eyfp* into. For ubiquitous expression of *granulito* in germ cells and somtic tissues, *granulito* was amplified using a forward primer containing mutations in the morpholino binding site (AAAGGATCCACCATGACGGAAGCTGAAGATCGGTGAATGAAGCTTG) and reverse primer (AAACTCGAGTTAGTCAGACAAGTGTTTGTAG) and cloned upstream of *globin*-3’UTR. Zebrafish *laminB2* (accession number NM_131002) was cloned from ovary cDNA using primers 5’AAAGGATCCATGGCGACCGCAACCCCGAGCCG3’ and

5’AAAGGATCCCACCATCACTGCACATTCTCTGG3’ and fused upstream to m*gfp* generating the construct pSP64T-*laminB2-mgfp-nos1*-3’UTR. To label germ cell granules we fused *vasa* N-terminal 369 amino acids of zebrafish Vasa, which are sufficient for localization to perinuclear granules [3], to dsRed Express at the C terminus; RNA contains the zebrafish *nos1*-3′UTR upstream to dsRed Express generating the construct pSP64T-*vasa-dsRedEx-nos1*-3’UTR. The zebrafish *NUPL1* (accession number NM_213229) was cloned from cDNA using primers 5’AGATCTATGTCTAGCTTTAACTTCGGCACAA3’ and

5’AGATCTGGCTCTCTTGCCCCTCTTGT3’. The ORF was then fused downstream to GFP generating the construct pSP64T-*mgfp-NUPL1-nos1*-3’UTR. Zebrafish *NUP155* was cloned from cDNA using the primers 5’GGATCCATGCCGTCCAGTCTGGGCTC3’ and 5’GGATCCTCAGTGCAGCTTCTCCAGCT3’ and cloned upstream GFP generating the construct pSP64T-*NUP155-mgfp-nos1*-3’UTR. Dynein light chain 2 like (accession number NM_001030000) was cloned from cDNA using primers 5’AGATCTATGACTGACAGGAAGGC3’ and 5’AGATCTTCAGCCCGATTTAAAG3’. The ORF was fused upsteam to EGFP to generate pSP64T-*Dyn2*-*egfp-nos1*-3’UTR. Zebrafish Dynamitin (genebank accession number DQ141218) was cloned from cDNA using the primers 5’GGATCCATGGCCGACCCGAAGTACG3’ 5’CTCGAGCTACTTGTTGAGTTTCTTCATCCTCTG3’. The ORF was then fused upstream *nos1 3’UTR* generating the construct pSP64T-*dynamitin*-*nos1*-3’UTR.

The constructs pSP64T-*H1M*-*egfp-nos1*-3’UTR, *H1M-mGFP-globin*-3’UTR [4], pSP64T-*egfp-farnesyl-nos1*-3’UTR, and pSP64T-*clip170-egfp-nos1*-3’UTR were used to label chromatin, plasma membrane and microtubules respectively. To inhibit cytokinesis, the mRNA of pSP64T-*N19RhoA-nos1*-3’UTR was injected. This dominant negative form of RhoA kindly provided by Michal Reichmann-Fried which was cloned by using primers 5’GCAGCCATCGTTGTCTTTTGGATCCTTTT3’, 5’CCTGTGGAAAGAACTGTTTGCTCA3’ 5’ TGAGCAAACAGTTCTTTCCACAGG3’ and ‘AAAACTCGAGATCTCCTTATAACAGCAGG3’, to exchange amino acid T19 with N19. For studying the subcellular localization of Tdrd7 protein, *Tdrd7* was amplified from cDNA (a mixture of ovary + mid-somites stages) with forward primer (AAAAGATCTAGGATGAGTGACGTGGAGTT) and reverse primer (AAATCTAGATAATACAACAAAACCTGAACACC) cloned in frame downstream of *egfp* into pSP64-*egfp-nos1*-3’UTR, replacing *nos1*-3’UTR with the *Tdrd7*-3’UTRits own.

***In situ hybridization for transcripts containing Tudor domains***

Zebrafish homologs of previously described tudor domain containing genes *RNF17*, *Tdrd1*, *Tdrd5*, *Tdrd6* and *Tdrd7* [5-9] at different stages starting from 4cell stage until 5 dpf were tested. One-colour whole-mount in situ hybridization was performed as previously described [10] with modifications described elsewhere [11, 12]. DIG-labeled antisense granulito-probe was synthesized using DIG nucleotide mix (Roche) and SP6-polymerase or T7-polymerase from a TOPO2 plasmid containing the gene of interest. *RNF17*, *Tdrd1*, *Tdrd5*, *Tdrd6* and *Tdrd7* were amplified from a mix containing ovary, mid somitogenesis and 3 dpf cDNA. *Tdrd7* (accession number EF643554). was amplified using primer (fw: 5’CGCATTAACGGCGAAAAA3’/rev: 5’GCAAACAAACCAAAGTGCAA3’). *Tdrd1* (XM_679932) was amplified with primers (fw: 5’TGTCTTGCAGTGGCACTTTC3’/rev: 5’AATTAACCCTCACTAAAGGGCAAGCAGGAGAACCAACTCC3’). *RNF17* (XM_692362.1) was amplified using primers (5’ACCAGCCCAAGTCAAACAAC3’/rev: 5’AATTAACCCTCACTAAAGGGAACACTGGTCTGGTGGAAGG3’). *Tdrd5* (XM_681163) was amplified using primers (fw: 5’CTGGTGTCAAAGCAACGAGA3’/rev: 5’AATTAACCCTCACTAAAGGGCCTGTTGGACTGGAAGTGGT3’). *Tdrd6* has in has 3 copies in the zebrafish genome (the probe for *Tdrd6* is a mixture of all 3), XM_687668 was amplified using primers (fw: 5’CCCATTCAGGCTGTTCAGTT3’/rev: 5’AATTAACCCTCACTAAAGGGTTTTCACCTGCTGCCTCTTT3’), XM_688932 was amplified using primers (fw: 5’GGTGCACAGCACGAGTTTTA3’/rev: 5’AATTAACCCTCACTAAAGGGTTTTTCACTCTCGGGCTCAT3’) and BX000362 was amplified using primers (fw: 5’GACCAATTTGGATCCACCAC3’/rev: 5’AATTAACCCTCACTAAAGGGAATGCAATGCAGAGCGTAA3’).

**References**

1. H Blaser, M Reichman-Fried, I Castanon, K Dumstrei, FL Marlow, K Kawakami, L Solnica-Krezel, CP Heisenberg, E Raz: **Migration of zebrafish primordial germ cells: a role for Myosin contraction and cytoplasmic flow**. *Dev Cell* 2006, **11**:613-27.

2. K Kawakami, H Takeda, N Kawakami, M Kobayashi, N Matsuda, M Mishina: **A transposon-mediated gene trap approach identifies developmentally regulated genes in zebrafish**. *Dev Cell* 2004, **7**:133-44.

3. U Wolke, G Weidinger, M Köprunner, E Raz: **Multiple levels of post-transcriptional control lead to germ line specific gene expression in the zebrafish**. *Curr Biol* 2002, **12**:289-294.

4. K Muller, C Thisse, B Thisse, E Raz: **Expression of a linker histone-like gene in the primordial germ cells in zebrafish**. *Mech Dev* 2002, **117**:253-7.

5. J Pan, M Goodheart, S Chuma, N Nakatsuji, DC Page, PJ Wang: **RNF17, a component of the mammalian germ cell nuage, is essential for spermiogenesis**. *Development (Cambridge, England)* 2005, **132**:4029-39.

6. S Chuma, M Hosokawa, K Kitamura, S Kasai, M Fujioka, M Hiyoshi, K Takamune, T Noce, N Nakatsuji: **Tdrd1/Mtr-1, a tudor-related gene, is essential for male germ-cell differentiation and nuage/germinal granule formation in mice**. *Proc Natl Acad Sci U S A* 2006, **103**:15894-9.

7. JM Smith, J Bowles, M Wilson, RD Teasdale, P Koopman: **Expression of the tudor-related gene Tdrd5 during development of the male germline in mice**. *Gene expression patterns* 2004, **4**:701-5.

8. M Hosokawa, M Shoji, K Kitamura, T Tanaka, T Noce, S Chuma, N Nakatsuji: **Tudor-related proteins TDRD1/MTR-1, TDRD6 and TDRD7/TRAP: domain composition, intracellular localization, and function in male germ cells in mice**. *Dev Biol* 2007, **301**:38-52.

9. T Hirose, M Kawabuchi, T Tamaru, N Okumura, K Nagai, M Okada: **Identification of tudor repeat associator with PCTAIRE 2 (Trap). A novel protein that interacts with the N-terminal domain of PCTAIRE 2 in rat brain**. *European journal of biochemistry / FEBS* 2000, **267**:2113-21.

10. T Jowett, L Lettice: **Whole-mount in situ hybridizations on zebrafish embryos using a mixture of digoxigenin- and fluorescein-labelled probes**. *Trends Genet* 1994, **10**:73-4.

11. G Hauptmann, T Gerster: **Two-color whole-mount in situ hybridization to vertebrate and Drosophila embryos**. *Trends Genet* 1994, **10**:266.

12. G Weidinger, U Wolke, M Koprunner, C Thisse, B Thisse, E Raz: **Regulation of zebrafish primordial germ cell migration by attraction towards an intermediate target**. *Development* 2002, **129**:25-36.
